# Structural insights into the binding mechanism of Clr4 methyltransferase to H3K9 methylated nucleosome

**DOI:** 10.1038/s41598-024-56248-2

**Published:** 2024-03-05

**Authors:** Christopher Saab, Joseph Stephan, Elias Akoury

**Affiliations:** 1 Department of Natural Sciences, Lebanese American University, Beirut, 1102-2801 Lebanon; 2 Department of Chemistry, McGill University, 801 Sherbrooke St. West, Montreal, QC H3AOB8 Canada; 3 School of Medicine, Lebanese American University, PO Box 36, Byblos, Lebanon

**Keywords:** Epigenetics, Chemistry

## Abstract

The establishment and maintenance of heterochromatin, a specific chromatin structure essential for genomic stability and regulation, rely on intricate interactions between chromatin-modifying enzymes and nucleosomal histone proteins. However, the precise trigger for these modifications remains unclear, thus highlighting the need for a deeper understanding of how methyltransferases facilitate histone methylation among others. Here, we investigate the molecular mechanisms underlying heterochromatin assembly by studying the interaction between the H3K9 methyltransferase Clr4 and H3K9-methylated nucleosomes. Using a combination of liquid-state nuclear magnetic resonance spectroscopy and cryo-electron microscopy, we elucidate the structural basis of Clr4 binding to H3K9-methylated nucleosomes. Our results reveal that Clr4 engages with nucleosomes through its chromodomain and disordered regions to promote de novo methylation. This study provides crucial insights into the molecular mechanisms governing heterochromatin formation by highlighting the significance of chromatin-modifying enzymes in genome regulation and disease pathology.

## Introduction

In eukaryotes, nuclear DNA associates with histone and non-histone proteins in nucleosomal arrays to assemble into chromatin fibers and form the chromosomes. The nucleosome array composes the chromatin fibers, where each unit consists of 146 base pairs of coiled DNA around an octamer of the histone proteins H2A, H2B, H3 and H4^1^. Triggered by high levels of folding, the diversified chromosomal processes highly depend on well-ordered structural chromatin organization. Heterochromatin is one of the unique structures in the nucleus and was first defined in plants as the element of the genome that preserves color stained DNA^2^. Until a decade ago, heterochromatin was assumed to be transcriptionally silent, but has ever since been known to play a significant responsibility in the regulation of gene expression, genomic stability, and chromosome transmission^3^. Notably, the structure–function relationship of heterochromatin is affected by the position effect variegation (PEV), where gene expression can be influenced by proximity to heterochromatic regions. Such genes may exhibit variegated expression patterns, leading to a mixture of active and silenced states that will eventually influence chromatin structure^4,5^. Although heterochromatin is characteristically gene-poor, its repetitive elements are marked with specific histone modifications that are distinct from the alterations in euchromatic or active genes. Two of these histone processes, acetylation/deacetylation, and histone-3-lysine 9 (H3K9) methylation, have been associated with different modes of heterochromatin assembly, gene regulation and inheritance^6,7^. These well-conserved ()modifications trigger high order heterochromatin packaging that form nucleation sites for recruiting DNA-binding proteins and RNA molecules. Histone methylation plays a major role in the pathogenesis of diabetes^8^ and cardiovascular diseases^9^ where many factors affect the levels of histone methyltransferases^10^, most importantly the coupling of histone methylation and DNA methylation^11^ in neurodevelopmental disorders^12,13^.

Though the mechanisms by which heterochromatin spreading and epigenetic inheritance remain unknown, the nucleation and assembly of the pericentromeric and telomeric heterochromatin are highly conserved from *Schizosaccharomyces* pombe (fission yeast) up to humans and require RNA interference (RNAi) machineries^14^. Studies in *S. pombe* have reported that heterochromatin spreading from nucleation sites depends on the presence of preexisting H3K9 trimethylation (H3K9me3) marks generated by the Clr4/Suv39h histone methyltransferase^15–17^. Importantly, the heterochromatic regions within the transcriptional silencing scheme depend on Heterochromatin protein 1 (HP1) proteins^18,19^. In several eukaryotes, HP1 paralogs (HP1α, β, and γ in humans; HP1a-e in *D. melanogaster*; Swi6 and Chp2 in S. pombe) have diverse functions and are required for heterochromatin maintenance, as they specifically bind H3K9-methylated (H3K9me) nucleosomes through their Chromodomain (CD)^20^.

**Figure 1:**
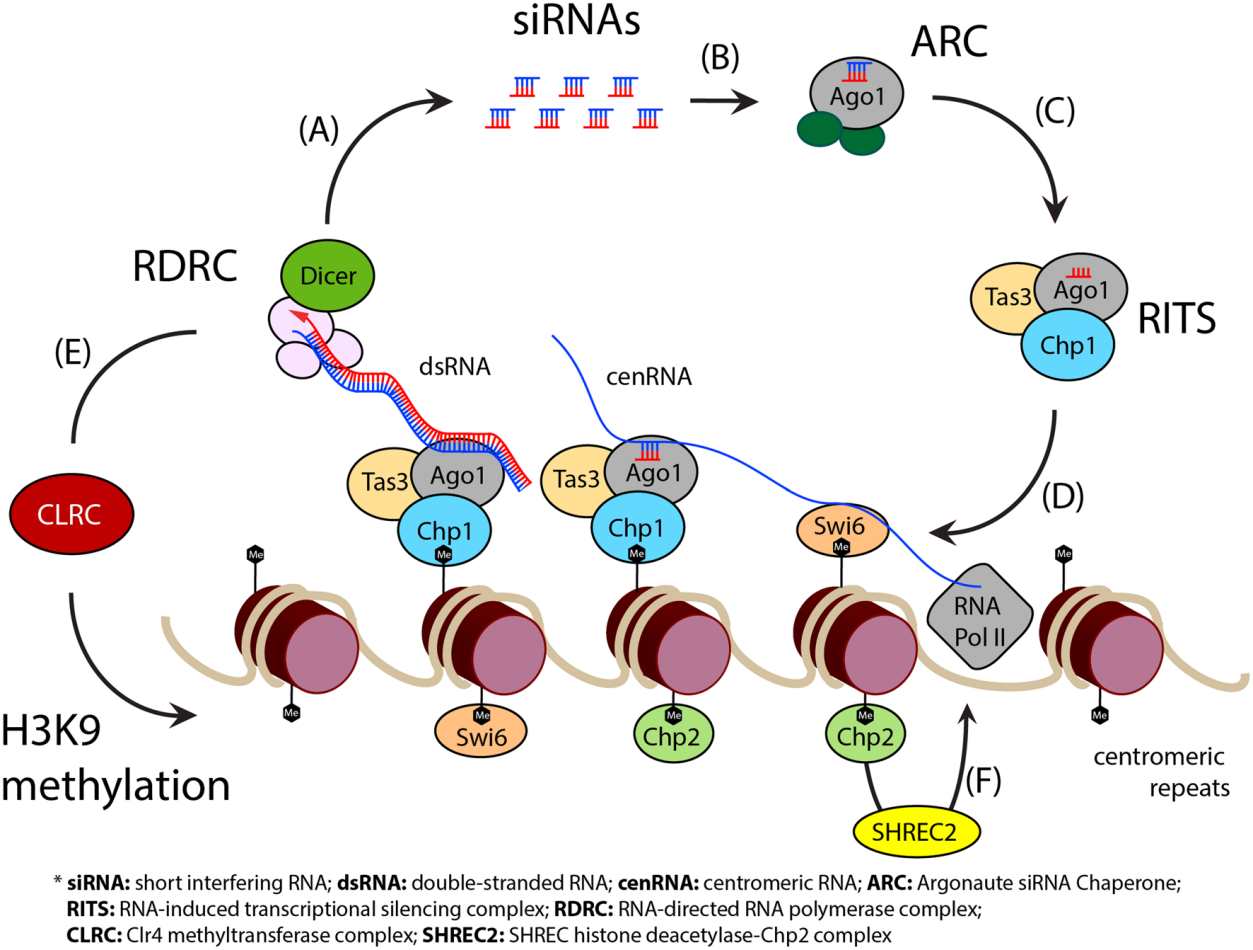
Heterochromatin formation in *Schizosaccharomyces pombe*. (**A**) The enzyme Dicer generates siRNAs from dsRNA. (**B**) siRNAs are subsequently loaded on Argonaute (Ago1). (**C**) Upon Ago1 interaction with Tas3 and Chp1, (**D**) the RITS complex associates with chromatin through base-pairing interactions between siRNAs and non-coding transcripts. This prompts the interaction of the Chp1’s chromodomain with H3K9 methylated nucleosomes further recruiting RDRC and Dicer to the centromeric repeats to synthesize new dsRNA and amplify siRNAs. This RNAi cycle promotes (**E**) RITS-directed recruitment of CLRC methyltransferase and enhances H3K9 methylation. Consequently, efficient silencing and heterochromatin formation require binding of the two HP1 proteins Swi6 and Chp2. Swi6 promotes further association of RITS with the non-coding RNA; and (**F**) Chp2 associates with the SHREC deacetylase complex to form SHREC2. Upon H3K14 deacetylation, SHREC2 mediates transcriptional gene silencing by limiting RNA polymerase II (Pol II) access to the heterochromatin. Note that SHREC consists of the four proteins Clr1, Clr2, Clr3 and Mit1.

At the molecular level, proper chromosomal segregation and deposition of centromere–specific nucleosomes require the establishment of RNAi-mediated heterochromatin^21^. Double-stranded RNA (dsRNA) generates small interfering RNAs (siRNAs) with the assistance of the Dicer enzyme and which are sequentially loaded onto the Argonaute (Ago1) protein for centromeric silencing (Fig. 1). siRNAs inactivate target RNAs by guiding the Ago1-containing RNA-Induced Transcriptional Silencing (RITS) complex to the corresponding centromeric transcripts^22^. Simultaneously, RNAi-mediated H3K9 methylation tethers to the chromatin through the interaction with Chp1, a Chromodomain protein of the RITS complex^22^. This process recruits the RNA-directed RNA polymerase complex (RDRC) to form new dsRNAs and consequently leads to siRNA amplification. In addition to the recruitment of RDRC, RITS targets the H3K9 Clr4-Rik1-Cul4 complex (CLRC) to the chromatin^23,24^. Importantly, Clr4 (Cryptic loci regulator protein 4), a lysine methyltransferase of the CLRC complex that is highly conserved between different species (Supplementary Fig. 1), subsequently marks the heterochromatin spread by H3K9 dimethylation (H3K9me2) and trimethylation (H3K9me3)^25^. These methylations allow further recruitment of HP1 paralogs, mainly Swi6 and Chp2^14^. Swi6 promotes the association of RITS with the non-coding RNA while Chp2 recruits the SHREC2 deacetylase complex that mediates transcriptional gene silencing^26^. In fission yeast, such transcription is repressed in regions close to the centromeres, telomeres, and silent mating-type cassettes by the proteins Chp1, Chp2, Swi6 and Clr4 all of which share a common structural domain, the chromatin organization modifier (chromo) domain^27^. Swi6 and Chp2 have two evolutionarily conserved domains, the N-terminal chromodomain (CD) and a C-terminal chromoshadow domain (CSD), connected by a hinge region (H) (Supplementary Fig. 2A)^28^. In fission yeast, Clr4 is characterized by the N-terminal CD and the C–terminal Su(var)3–9 Enhancer of zeste Trithorax (SET) domain located between the pre-SET and the post-SET domains. The CD is found in many chromosomal proteins and folds to generate a binding site for the N-terminal tails of histone proteins.

The CD specifically recognizes H3K9 methylated histones and recruits other CD proteins to these sites. This recruitment, prompted by high levels of H3K9 methylation and siRNAs at initiation sites, marks heterochromatin formation and spreading into neighboring regions^29^. In parallel, Swi6 and Chp2 dimerize through their CSD to form a peptide-binding surface^14^. (Supplementary Fig. 2B). Additionally, the chromo shadow and hinge domains interact with the histone H3K9 methyltransferase Clr4 to tether the latter to the locus for further assistance in heterochromatin spreading^30^. HP1 proteins also mediate the recruitment of chromatin-modifying factors such as the histone SHREC2 deacetylase complex. The cryptic loci regulator proteins Clr1, Clr2 and Clr3 regulate mating-type silencing (Supplementary Fig. 2C) and facilitate histone modifications and nucleosome positioning to organize higher-order chromatin structure^31,32^. Therefore, HP1 proteins provide a foundation for heterochromatin self-assembly and spreading through interactions with both histone-modifying enzymes and methylated histones. This reorganization of the chromatin structure is essential for silencing transcription, suppressing recombination and maintaining genomic integrity^14^.

Despite extensive biochemical and genetic studies aimed at revealing the mechanisms by which Clr4 contributes to the formation of higher-order chromatin structures, and catalyzes the trimethylation of H3K9, the exact molecular mechanisms by which Clr4 contributes to the establishment and propagation of heterochromatin domains are still not fully understood. Further research is needed to uncover the specific functions and interactions of Clr4 in heterochromatin assembly and maintenance.

Specifically, and while accumulating studies have revealed its diverse biochemical and cellular functions^33,34^, the mechanistic contributions and structural characteristics of the distinctive domains remain obscure. On the other hand, despite multiple reported NMR and crystal structures of isolated CD proteins bound to H3K9me peptides^35–37^, the exact mechanisms by which these proteins interact with their actual full-sized H3K9me nucleosome binding partner and contribute to the dissemination of chromatin structure remains unsolved. Here we study the interaction of Clr4 with H3K9 methylated nucleosomes using a combination of liquid-state NMR spectroscopy and cryo-EM to reveal structural insights into the binding mechanism of Clr4 to the H3K9 methylated nucleosome.

## Materials and methods

### Recombinant protein expression and purification

All plasmids, oligonucleotides and protein tags used in the study are reported in Supplementary Table 1. The Clr4 full-length plasmid was cloned in a pET30a expression vector encompassing an N-terminal His-tag and a C-terminal FLAG-tag. Clr4 constructs were then generated by inverse Polymerase Chain Reaction (PCR) using the Clr4 full-length plasmid. Briefly, the amplification of target DNA fragments was accomplished using designed primers (forward and reverse, reported in Supplementary Table 1) to introduce modifications or deletions in the Clr4 sequence. For each construct, the DNA fragment was purified and ligated into a pET30a cloning vector. The ligated plasmids were then transformed into competent *Escherichia coli* (*E. coli)* cells. Positive clones were identified through colony PCR screening and confirmed by DNA sequencing. Finally, plasmids carrying the verified constructs were extracted from the bacterial colonies and purified for protein expression and purification. Each construct was then overexpressed in *E. coli* Bl21(DE3) pLysS cells and purified by affinity chromatography (GE Healthcare Freiburg, Germany) as previously reported^38^. Briefly, protein expression was induced by 0.5 mM IPTG and cell cultures were cultivated for 18 h at 18 °C. *E. coli* Bl21(DE3) pLysS cells were grown in 6 L of M9 minimal medium containing ^14^N or ^15^N-NH_4_Cl and ^12^C or ^13^C-Glucose depending on the construct (unlabeled, ^15^N- or ^15^N/^13^C-uniformly labeled proteins). After harvesting, cells were re-suspended in lysis buffer (50 mM HEPES pH 7.5, 150 mM NaCl, 3 mM beta-mercaptoethanol, 20 mM imidazole) and flash-frozen for storage at -80ºC. During purification, cells were incubated on ice for 30 min in lysozyme before sonication (Branson Sonifier 250-output 4, duty cycle 40). After ultracentrifugation at 12,000*g* for 30 min at 4ºC, the supernatant was incubated for 30 min at 4 °C with the binding buffer (50 mM HEPES pH 7.5, 500 mM NaCl, 3 mM beta-mercaptoethanol, 20 mM imidazole) on Ni–NTA resin (GE Healthcare, Freiburg, Germany). Protein elution from the resin was accomplished by using the elution buffer (50 mM HEPES pH 7.5, 500 mM NaCl, 3 mM beta-mercaptoethanol, 300 mM imidazole). After dialysis in 50 mM HEPES pH 7.5, 150 mM NaCl, and 3 mM beta-mercaptoethanol, constructs were further purified by size exclusion chromatography on a Superdex 200 column (GE Healthcare) and were stored in the same buffer for cryo-EM analysis. Otherwise, ^15^N- and ^15^N/^13^C-uniformly labeled proteins were dialyzed in a buffer containing 50 mM phosphate buffer pH 6.8, 150 mM NaCl, 1 mM Dithiothreitol (DTT), and were concentrated by centrifugal filtration for NMR analysis.

### Histone protein expression and in vitro nucleosome reconstitution

The four Xenopus laevis histone proteins H2A, H2B, H3 and H4 were recombinantly overexpressed in *Escherichia coli* according to protocol^39^. H3K9 was methylated by applying the Methyl Lysine Analog (MLA) method^40^ as described in Supplementary Fig. 3 to mimic H3K9 methylation. Briefly, MLA histone 3 was obtained upon alkylating a cysteine residue to a trimethyl and is therefore termed H3KC9me3. The presence of MLA product was confirmed with mass spectrometric analysis (Supplementary Fig. 3). DNA was PCR amplified from a plasmid containing the Widom 601 DNA sequence and purified by phenol–chloroform extraction. After ethanol precipitation, the DNA was resuspended in 20 mM HEPES/NaOH pH 7.5, 2 M NaCl, 1 mM DTT. The histone octamer was then equilibrated in 20 mM HEPES pH 7.5, 1 M NaCl, 1 mM DTT and subsequently purified by size exclusion chromatography. Nucleosome reconstitution^41^ was performed after mixing the histone octamer:DNA ratio in dialysis bags immersed into a 1 L of buffer containing 20 mM HEPES/NaOH pH 7.5, 1 M NaCl, 1 mM DTT and dialyzed for 12 h at 4 °C. After performing dialysis twice, the dialysis buffer was replaced with 1 L low salt buffer (20 mM HEPES/NaOH pH 7.5, 75 mM NaCl, 1 mM DTT) and dialyzed for 12 h at 4 °C. The reconstituted nucleosomes were concentrated to 2 mg/ml in a buffer containing 20 mM HEPES pH 7.5, 75 mM NaCl, 1 mM DTT and were analyzed on 5% native PAGE.

### Assembly and pulldown assay of Clr4-H3KC9me3 nucleosomes

The pulldown assay of Clr4 constructs (Clr4 FL, CD, DCD, Pre-SET-Post, SET-Post) with H3KC9 nucleosomes was performed using the following protocol: 0.5 mg of protein was first added to 15 ml of anti-FLAG M2 affinity gel resin (Sigma-Aldrich) and incubated in the binding buffer (50 mM phosphate buffer pH 6.8, 150 mM NaCl, 1 mM DTT) for 20 min at 4 °C. After washing the resin with the binding buffer and incubating 2 mg of nucleosomes in a volume of 40 ml for 1 h on ice, the flow-through was collected after centrifugation. The flowthrough, resins and inputs were analyzed on silver stained (0.1% AgNO_3_) SDS-PAGE 15% acrylamide gel and western blot using anti-H3K9me3 antibody (AbCam, 1:1000). Clr4-H3KC9me3 nucleosome complexes were then assembled using 5 mg protein and 10 mg nucleosomes in 50 mM phosphate buffer pH 6.8, 150 mM NaCl, 1 mM DTT and eluted after incubation for 20 min with FLAG peptide (Sigma Aldrich) at 4 °C. The complexes were then evaluated on 15% acrylamide SDS-PAGE gels and by negative stain Electron Microscopy.

### Microscale thermophoresis (MST)

100 μg of Clr4 protein constructs were fluorescently labeled using the MO-L003 Monolith Protein Labeling Kit BLUE-NHS (Amine reactive—Nano temper Technologies, Munich, Germany) in a 1:1 fluorescence: protein ratio.1 300 ng of fluorescent Clr4 constructs were prepared in 20 μl buffer containing 10 mM Tris–HCl pH 7.5, 150 mM NaCl, 0.5 mM DTT, 0.05% Tween-20 and H3KC9me3 nucleosomes were added in increasing concentrations 8 nM, 30 nM, 65 nM, 120 nM, 210 nM, 350 nM, 450 nM, 650 nM, 760 nM, 870 nM, 1.5 μM, and 2.5 μM. MST measurements were acquired using Nanotemper 115 Monolith instrument with standard treated capillaries (Nano temper Technologies, Munich, Germany) using 30 s on-time laser and 5 s off-time laser with 40% MST power and 80% LED. Five runs were performed per measurement. Data were analyzed with GraphPad Prism (San Diego, CA, USA) and the sigmoid trend was fitted with Richard’s five parameter logistic asymmetric sigmoidal equation for the automatic calculation of K_d_.

### NMR spectroscopy

All NMR experiments were recorded at 288 K on Bruker Avance III 800 MHz spectrometer equipped with a cryogenic probe. 1 mM protein of each labeled construct (^15^N/^13^C- Clr4 CD, Clr4 1–191 and Clr4 192–490) was prepared in 50 mM phosphate buffer pH 6.8, 150 mM NaCl, 1 mM DTT, 10% (v/v) D_2_O. Three-dimensional HNCA, HNCACB, HN(CO)CACB and HNCO triple-resonance experiments^42^ and two-dimensional HSQC spectra were acquired for the NMR backbone assignments of all constructs. 3D HNCACB and 3D HNCA spectra were recorded with 2048 (F1) × 84 (F2) × 128 (F3) complex points, 24 scans per increment with spectral widths of 7716 Hz, 1705 Hz and 10,582 Hz in the ^1^H, ^15^N, ^13^C dimensions, respectively, and a total experiment time of 3 days. 3D HNCO spectra were recorded with 2048 (F1) × 100 (F2) × 100 (F3) complex points, 8 scans per increment with spectral widths of 7003 Hz, 1561 Hz and 1233 Hz in the ^1^H, ^15^N, ^13^C dimensions, respectively, and a total experiment time of 3 days. 2D ^1^H-^15^N HSQC spectra were acquired using 600 complex points and 32 scans per increment with spectral widths of 8389 Hz and 1844 Hz in the ^1^H and ^15^N dimensions, respectively, and a total experiment time of 9 h. NMR titrations between Clr4 constructs and H3KC9me3 nucleosome were completed on samples containing 1 mM ^15^N-labeled protein (Clr4 1–191 or Clr4 192–490 construct) in 50 mM phosphate buffer pH 6.8, 150 mM NaCl, 1 mM DTT, 10% (v/v) D_2_O; and were freshly prepared in 1:0.5 ratio. All NMR experiments were processed with NMRPipe^43^ and analyzed with CCPN Analysis^44^. The intensity ratio plots are reported with a 3-residues averaging window.

### Transmission electron microscopy

To determine the structure of the Clr4–H3KC9me3 complex using cryo-EM, we first encased Full-Length (FL) Clr4 in a uranyl acetate negative stain to evaluate the stability of the complex. Negative stain EM grids were prepared after spotting 4 μl of protein samples for 30 s on a glow discharged copper grid (Cu 400 mesh Q11916, Quantifoil Micro tools, Grossloebichau, Germany) coated with 1 nm carbon film followed by 15 s incubation in 2% Uranyl acetate. Negative stain images were inspected on a Morgani transmission electron microscope (FEI Company, Hillsboro, USA) to evaluate the grid homogeneity.

### Cryogenic electron microscopy

Cryo-EM grids were prepared as previously described^45^. Briefly, 0.5 mg/ml Clr4–H3KC9me3 complex was diluted and 4 μl were applied to glow discharged cryo-EM Holey carbon–coated grids (Cu 300 mesh R3/3 + 1 nm carbon layer, Quantifoil Micro tools, Grossloebichau, Germany). Grids were then blotted for 3 s and plunge-frozen in liquid ethane using Vitrobot Mark IV (FEI Company, Hillsboro, USA). Low dose (15e^–^/Å^2^) projection images were collected using a Tecnai Spirit transmission electron microscope (FEI Company, Hillsboro, USA) at accelerating voltage of 200 kV cooled at liquid nitrogen temperature to inspect the grid quality at 59,000 × magnification. Cryo-EM data were then collected using a Titan-Krios transmission electron microscope (FEI Company, Hillsboro, USA) at 300 kV and 113,000 × magnification using F816 CMOS camera (TVIPS GmbH, Gauting, Germany) with an image pixel size of 1.2 Å per pixel on the object scale. Automated data collection was recorded with EM-TOOLS (TVIPS GmbH, Gauting, Germany) in defocus ranging between 10,000 Å and 40,000 Å. Single-particle analysis was performed on 2480 micrographs of Clr4–H3KC9me3 nucleosome complex and on 1025 micrographs of H3KC9me3 reference nucleosome using XMIPP software package^46^ and extensively refined with SPIDER^47^ to reach the best reconstruction at 3.8 Å resolution. After data collection, single particles were selected manually and then semi-automatically from 2D projections; particle alignment and 3D de novo reconstructions were performed. The 3D reconstruction from cryo-EM images requires averaging of 10,000 to 1,000,000 single images. Therefore, the assembled complex must preferably be in one or only a few defined conformations. However, interactions are transient if proteins are flexible, which reduces the chance of solving the structure at high resolution using cryo-EM. We first optimized the binding ratios between Clr4 and H3KC9me3 nucleosomes using negative stain EM to select the most stable complex prior to recording cryo-EM micrographs. Though the individual particles are imaged at low contrast, the consequential structure can be of extremely high resolution after averaging hundreds to thousands of particles. 2480 cryo-EM micrographs were then collected and displayed as single particles of the Clr4-H3KC9me3 nucleosome complex in different orientations. We generated 2D class averages with RELION and 3D refinements with SPIDER. Initial rigid body docking and molecular models were built with ChimeraX^48^ using rigid body fitting of the Clr4 CD (PDB 1G6Z), Clr4 SET-Post domain (PDB 1MVH), and nucleosome (PDB 1AOI). The fit into the density was automatically performed with ChimeraX with minor manual adjustments.

## Results and discussion

**Figure 2:**
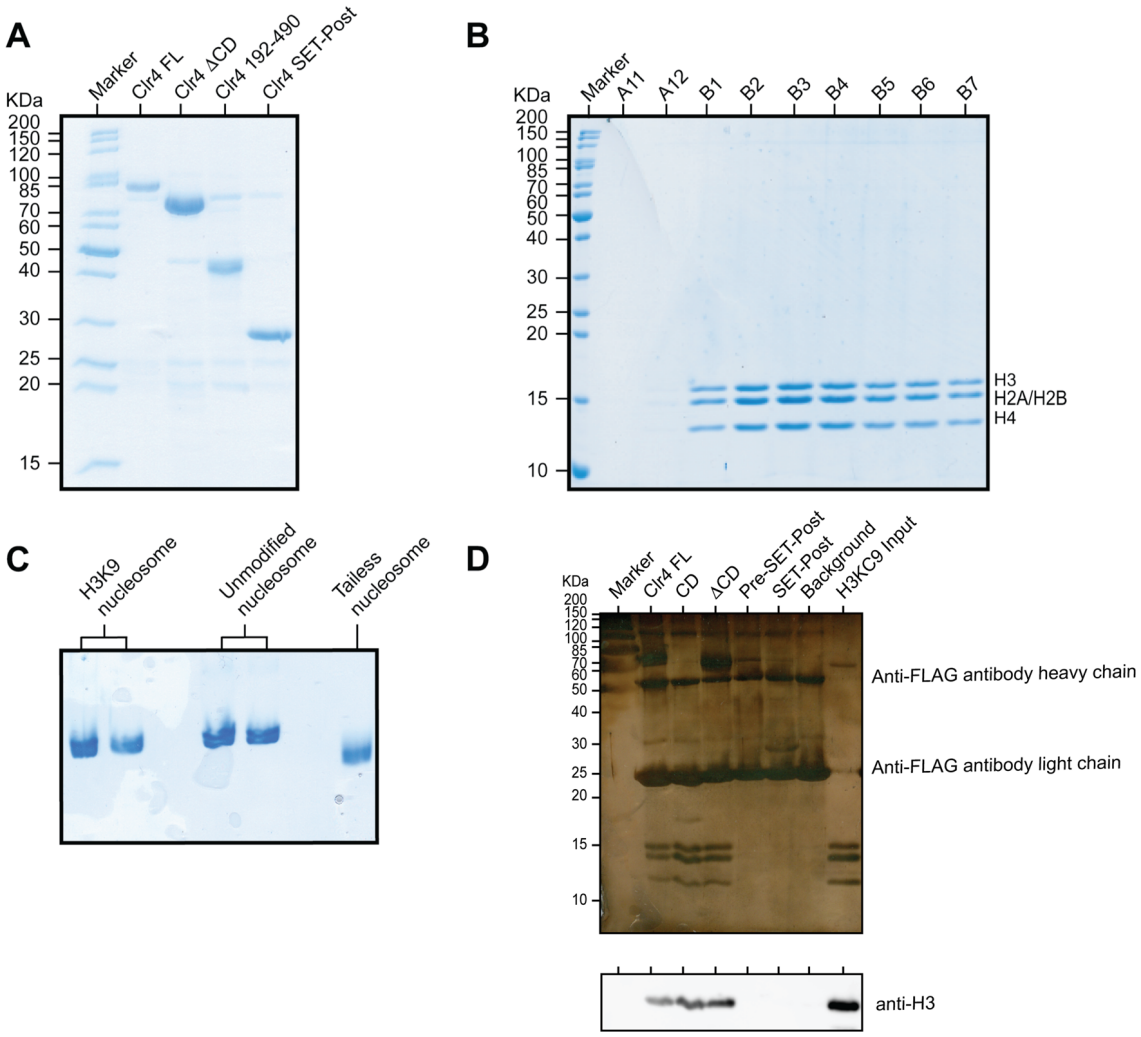
Assembly of FL Clr4-H3KC9me3 Methylated Nucleosome Complex. (**A**) Coomassie-stained SDS-PAGE of purified N-terminal His-tagged and C-terminal FLAG-tagged protein constructs: Clr4 FL, ΔCD, 192–490, and SET-post. (**B**) Coomassie-stained 15% SDS-PAGE of purified H3, H2A, H2B and H4 histone proteins from FPLC-purified fractions A11-B7. (**C**) Native gel showing the assembly of methylated, unmodified, and tailless nucleosomes. (**D**) In vitro pulldown assay using silver stained SDS-PAGE and western blot analysis of the interaction between Clr4 constructs and H3KC9me3. Clr4 constructs were bound to FLAG resin, incubated with H3KC9me3 nucleosome, eluted from the resin and detected with anti-H3K9me3 antibody, demonstrating the necessity of the Clr4 disordered region for the formation of the Clr4-H3KC9me3 nucleosome complex. The full-length membranes of all gels are reported in Supplementary Fig. 3F.

We previously showed that the CD and the disordered region, connecting the CD with the SET domain, both bind the nucleosome core and contribute to H3KC9 methylation in vitro^38^, thus confirming that the disordered region stabilizes Clr4 interaction with the unmodified nucleosome to deposit the initial H3KC9 methylation. In this study, we extended our previous work to incorporate structural insights into the binding mechanism of full-length Clr4 to the H3KC9 methylated nucleosome. We overexpressed full-length Clr4 protein and several domains (CD, CD-deleted construct (ΔCD), residues 1–191, residues 192–490, and SET-Post domains) in E *coli* then purified the constructs following optimized protocols^38^ (Fig. 2A). Histone proteins H2A, H2B, H3 and H4 were expressed and purified prior to in vitro nucleosome reconstitution as detailed in the methods section (Fig. 2B,C). It is important to mention that H3K9 is methylated by applying the Methyl Lysine Analog (MLA) method^40^ to alkylate a unique cysteine residue by a methylating agent to mimic the trimethylation of lysine 9 on histone 3. A chemical modification occurs whereby the lysine is mutated to a cysteine, which is then alkylated to a trimethyl moiety, hence the term H3KC9me3. (Supplementary Fig. 3). We first evaluated severalClr4 constructs containing a C terminal FLAG-tag for binding to the methylated H3KC9me3 nucleosome using pull-down assays and anti-FLAG resin. After washing unbound nucleosomes and running silver-stained SDS-PAGE and western blot gel electrophoresis, we confirmed that full-length Clr4, CD and ΔCD constructs bind to H3KC9me3 nucleosome (Fig. 2D). This was in line with our previous study that highlighted the vital role of the disordered region in Clr4 binding to the nucleosome using pull-down assays with constructs comprising residues 1–191 and 192–490; and mutational analysis in the Clr4 disordered region (^164^QKRELVS^170^ to ^164^SGSGSGS^170^ and ^147^TNSK^150^ to ^147^SGSG^150^) that showed reduced Clr4 interaction with the nucleosome and its methyltransferase activity^30^. We then highlighted the structural role of the disordered (S69 to P191) and ordered (S192 to G490) regions upon binding to the H3KC9me3 nucleosome. Clr4 displays 3 major stretches with a high disorder probability between S69 to T130, and a moderate probability between S178 and P191 (Supplementary Fig. 4). We showed that de novo H3KC9 methylation is formed by nonspecific interactions between the nucleosome and the disordered region, underlining the crucial role played by these disordered stretches in the spread and establishment of heterochromatin. These structural insights into Clr4 methyltransferase binding to the H3KC9 methylated nucleosome via Clr4’s disordered regions are vital to furthering our understanding of heterochromatin. In *S. pombe*, the CLR4 gene encodes a 490-residue protein Clr4 which is an ortholog to the human SUV39H1protein^49^. Residues Y8–S69 and residues L328–A452 constitute to the CD and SET domains of Clr4, respectively. The SET domain is bordered by the pre-SET (S258–L328) and the post-SET (L473–G490) domains (Supplementary Fig. 4A & B). The CD comprises three beta-strands and one alpha-helix that recognizes the H3KC9 methylated site through an aromatic cage^50^. This interaction determines how the CD coordinates their distinct functions at the same locus. On the other hand, residues 69–191 are within a highly disordered region whereas the methyltransferase catalytic core is incorporated within the SET domain of Clr4 where it specifically methylates lysine 9 of histone H3. The structure of the SET domain is rich with beta-strands and several loops. The pre-SET domain incorporates 9 cysteines that form a triangular zinc cluster, whereas the post-SET domain remains largely unstructured (Supplementary Fig. 4)^51^. Many studies highlighted that H3K9 methylation alters the way CD interacts with the nucleosome, yet the precise mechanism by which H3K9 methylation modifies this interaction, the potential role of the highly unstructured motif between residues S69 and S258; and the structural/functional impact of the full-length Clr4 on the H3K9 nucleosome all remain undetermined.

### Cryo-EM structure of Clr4-H3KC9me3 nucleosome complex

**Figure 3:**
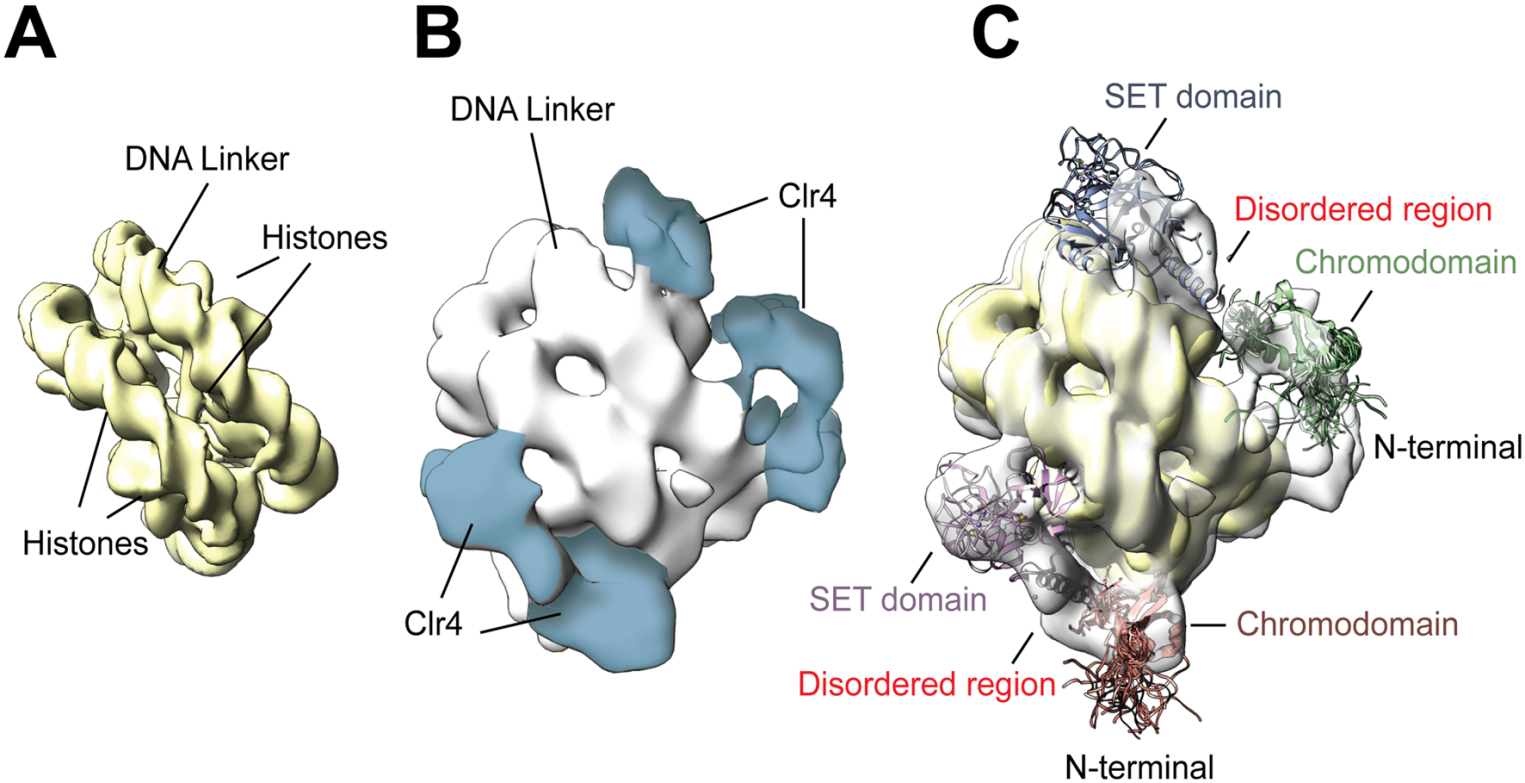
Overview of The Clr4-Nucleosome Structure. (**A**) Cryo-EM maps of Clr4 methyltransferase in its (A) unbound (3.8 Å) and (B) bound form to H3KC9me3 nucleosomes (4.5 Å). (**C**) Both maps are overlaid to confirm the additional densities corresponding to Clr4 interaction with H3KC9me3 nucleosomes. Docking of the Clr4 Chromodomain NMR solution structure (PDB 1G6Z yellow) and the crystal structure of Clr4 Pre-SET-post domains (PDB 1MVH, green) into the cryo-EM Clr4 FL-H3KC9me3 nucleosome complex map. Cryo-EM data collection, structure refinement and validation statistics are reported in Supplementary Table 2.

No structural determination of full Length Clr4 in free or nucleosome-bound forms exists to date, due to the complex’s large size and inherent disorder, as well as technical limitations. We used the combination of NMR and cryo-EM to improve structure resolution of Clr4-H3KC9me3 nucleosome. We first optimized the binding ratios between Clr4 and H3KC9me3 nucleosomes using negative stain EM to select the most stable complex prior to recording cryo-EM micrographs. After data collection of the Clr4-bound H3KC9me3 nucleosome complex at cryogenic temperatures (Supplementary Fig. 5), cryo-EM micrographs were displayed as single particles of the Clr4-H3KC9me3 nucleosome complex in different orientations. For a projection-matching approach, we used the cryo-EM map of the free H3KC9me3 nucleosome control as a template for alignment. From a total of 1,200,000 particles, we classified H3KC9me3 nucleosomes into 6 classes (Supplementary Fig. 6A). Class N_1_ contains 51% of all particles and shows the core nucleosome particle. Classes N_2_–N_6_ show moderate to weak densities of the core nucleosome particle. After initial reconstructions and remodeling, the free H3KC9me3 nucleosome was refined to 3.8 Å (Fig. 3A) while the Clr4-H3KC9me3 nucleosome complex was refined to 4.5 Å (Fig. 3B,C and Supplementary Fig. 6B-E). Cryo-EM data collection, structure refinement and validation statistics are reported in Supplementary Table 2.

### Clr4 binds H3KC9me3 nucleosomes through its domains and disordered region

**Figure 4:**
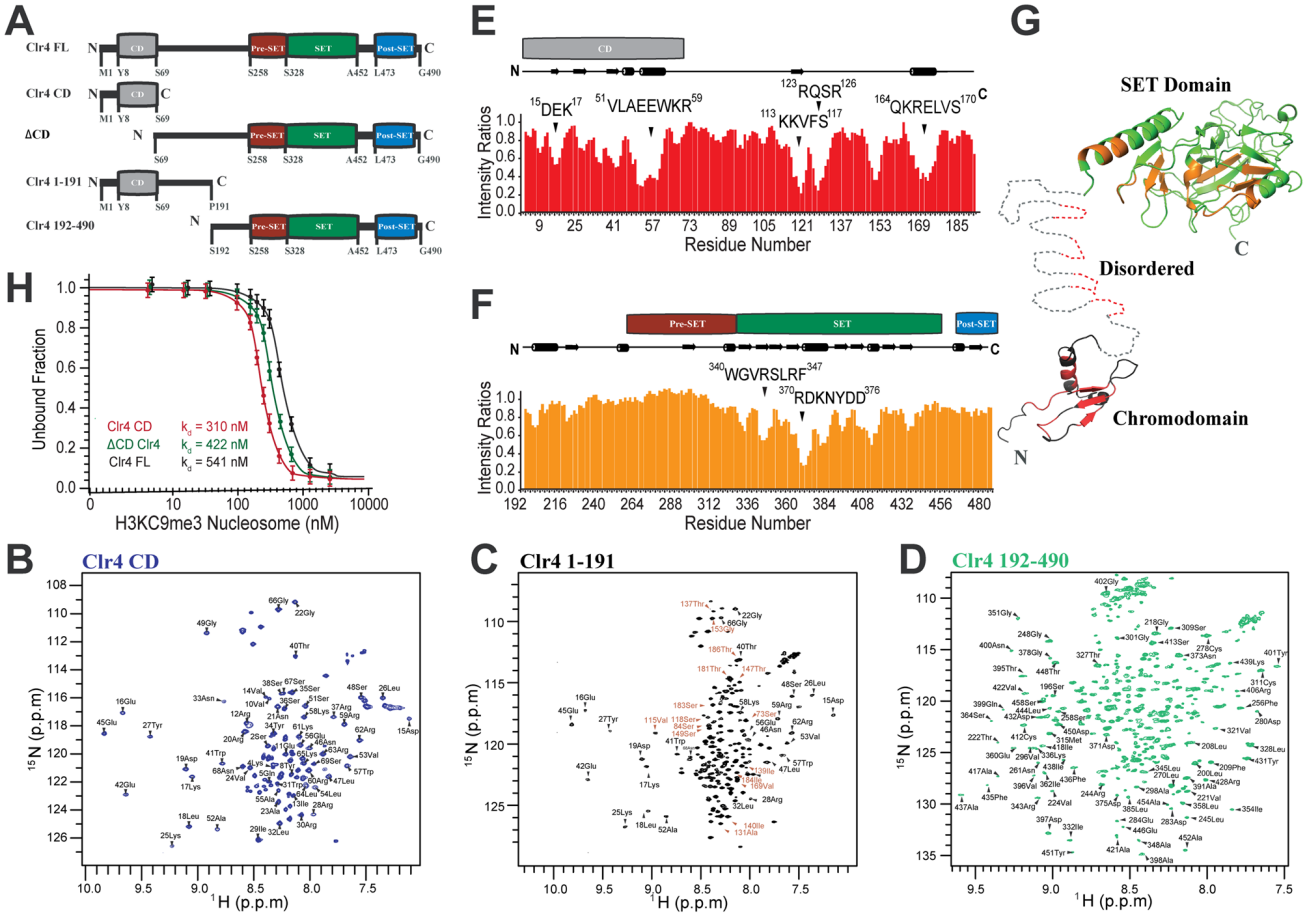
Deciphering the Interaction of Clr4-H3KC9me3 Methylated Nucleosome Complex by NMR. (**A**) A schematic diagram representing the domain organization of FL Clr4 protein, CD, ΔCD, 1–191 and 192–490 constructs (N = N-terminal, C = C-terminal, CD = Chromodomain, SET = SET domain, FL = Full length). The numbering reflects the primary sequence of the protein. Two-dimensional ^1^H-^15^N HSQC spectra of isotope-labeled (**B**) Clr4 CD, (**C**) 1–191^40^ and (**D**) 192–490 constructs with backbone amide assignments. For visualization purposes, only selected assignments are shown in (C) and (D) and the full chemical shift assignment is reported in Supplementary Table 3. The shifts in red (spectrum C) belong to the disordered part of Clr4 (residues 70 to 191). Spectra were recorded on a Bruker Avance III 800 MHz spectrometer at 288 K. Intensity ratio plots of E) Clr4 1–191 and (D) Clr4 192–490 in the presence of H3KC9me3 nucleosome (1:0.5 ratio). Intensity ratios represent the backbone amide resonances with the residue patches of the chromodomain, disordered regions, and Set domain showing the most significant decrease in signal intensities in presence of H3KC9me3 nucleosomes (detailed in Supplementary Fig. 7). The secondary structure elements of Clr4 are represented. (G) Based on the intensity ratio plots, the binding sites of H3KC9me3 nucleosome are mapped on the Clr4 CD (PDB 1G6Z, in red), on the disordered regions and on the SET domain (PDB MVH1 in orange). (H) Microscale Thermophoresis assay reporting the binding curves of FL Clr4, ΔCD and CD with the dissociation constants (K_d_) of 541 nM, 422 nM and 310 nM, respectively.

Next, we mapped the Clr4-H3KC9me3 nucleosome binding sites using liquid-state NMR spectroscopy by acquiring 2D heteronuclear single quantum coherence (HSQC) spectra of ^15^N-labeled Clr4 CD, ^15^N-labeled Clr4 1–191 and ^15^N-labeled Clr4 192–490 constructs (Fig. 4A-D). After recording the NMR experiments for protein assignments (HNCA, HNCACB, HN(CO)CACB and HNCO) for the three constructs, we assigned the backbone resonances to a high degree of completeness after confirming the ^1^H and ^15^N assignments for 448 of the 466 assignable amide groups (96.0%), and the ^13^C assignments for C’, Cα and Cβ nuclei (95.1%, 97.8% and 96.2%, respectively). The chemical shift assignments of FL Clr4 have been deposited in the Biological Magnetic Resonance Bank BMRB and reported in Supplementary Table 3. We then acquired 2D HSQC spectra of ^15^N-labeled Clr4 1–191 and Clr4 192–490 in free and bound forms to the H3KC9me3 nucleosomes to follow the changes in chemical shifts and intensities of individual peaks (Supplementary Fig. 7A-B). The analysis of NMR intensity ratios between bound and free forms with respect to the Clr4 residue number is a direct indication of binding where signal broadening (ratio < 1) is the result of exchange of the protein between the free and bound conformations. The interaction of Clr4 with the H3KC9me3 nucleosome implicated residue patches from the CD (^8^YEVER^12,15^DEK^17^, ^25^KLYR^28^, ^31^WLNY^34^, ^40^TWE^41^, ^51^VLAEEWKR^59^), from the disordered region (^113^KKVFS^117^, ^123^RQSR^126^, ^147^TNSK^150^, ^164^QKRELVS^170^, ^196^SYT^198^, ^201^SFY^203^, ^227^VDDE^230^) and the SET domain (^330^LEIFK^334^, ^340^WGVRSLRF^347^, ^370^RDKNYDD^376^, ^381^YLFD^384^, ^394^YTV^396^, ^405^SRFFNH^410^, ^431^YDLAFF^436^) (Fig. 4E-F and Supplementary Fig. 7C-D). These Clr4-H3KC9me3 nucleosome binding interfaces were highlighted on the 3D structure of the protein’s chromodomain, SET domain and the disordered regions (Fig. 4G). We have previously reported that the Clr4 disordered region between residues 69 and 191 stabilizes the Clr4-H3KC9me3 complex and tethers the CD to the nucleosome^38^. Also in the same study, we showed that the propensity for secondary structure elements becomes visible at this intrinsically disordered region upon Clr4-H3KC9me3 complex formation where a beta-strand at ^113^KKVFS^117^ and an alpha-helix at ^164^QKRELVS^174^ emerged^38^. In the current study, we are expanding our understanding of the SET domain, which plays a role in determining the methylation levels of lysine 9 of histone H3, regulation of the chromatin function and epigenetic control of gene function^38,52^. As observed for several SET domain-containing proteins, the pre-SET domain of Clr4 (residues S258-S328) encloses 9 conserved cysteine residues that coordinate 3 zinc ions to structurally stabilize the two patches of random coils^53^. Similarly, 3 conserved cysteines in the disordered post-SET domain (residues L473-G490) form a zinc-binding site with a cysteine located in the SET domain active site^51^. The SET domain (residues S328-A452) consists of 8 beta-strands, 3 alpha-helices and several loops.

Even though NMR titration experiments are routinely performed to study molecular interactions and determine the dissociation constant (K_d_) between two protein partners, this approach suffers some limitations. For instance, relatively high concentrations and different ratios of both partners are required to decipher K_d_ properly. Other drawbacks lie in overlapping peaks and line broadening, mostly encountered in biomolecular studies of large systems. Since both were observed during our Clr4 and H3KC9me3 nucleosome interactions at high concentrations and ratios, we refrained from using NMR and instead applied a Microscale Thermophoresis (MST) assay to report K_d_ values from the binding curves of Clr4 constructs (Fig. 4H). Upon increasing concentrations of H3KC9me3 nucleosome, full length Clr4, ΔCD and CD showed K_d_ values of 541 nM, 422 nM and 310 nM, respectively. Taken together, our results suggest that CD binding to the H3KC9me3 nucleosome is strengthened by the interaction of the disordered regions with the nucleosome, which then allows further binding of the SET domain.

### Structure of the Clr4-H3KC9me3 nucleosome complex

**Figure 5:**
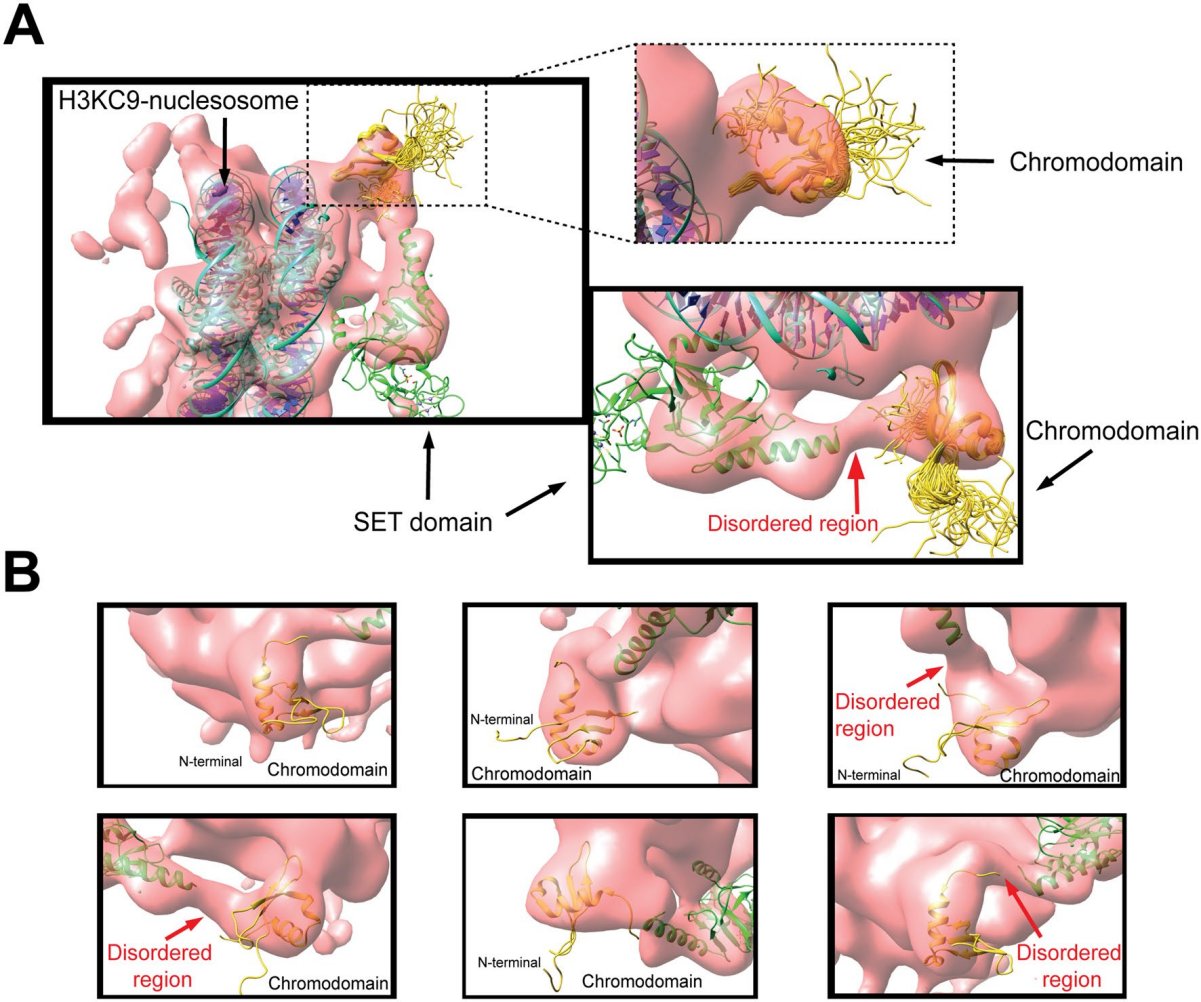
Cryo-EM Reconstruction of FL Clr4–H3KC9me3 Nucleosome Complex. (**A**) Molecular docking of the H3K9 nucleosome crystal structure (PDB 1AOI), the Clr4 CD NMR solution structure (PDB 1G6Z yellow) and the crystal structure of Clr4 Pre-SET-post domains (PDB 1MVH, green) into the cryo-EM Clr4 FL-H3KC9me3 nucleosome complex map. (**B**) Docking of the corresponding secondary structures reveals an extra density (indicated by red arrow) which explains the interactions of the Clr4 1–191 and Clr4 192–490 constructs with the core of H3KC9me3 methylated nucleosome as accessed by NMR spectroscopy. Cryo-EM data collection, structure refinement and validation statistics are reported in Supplementary Table 2.

**Figure 6:**
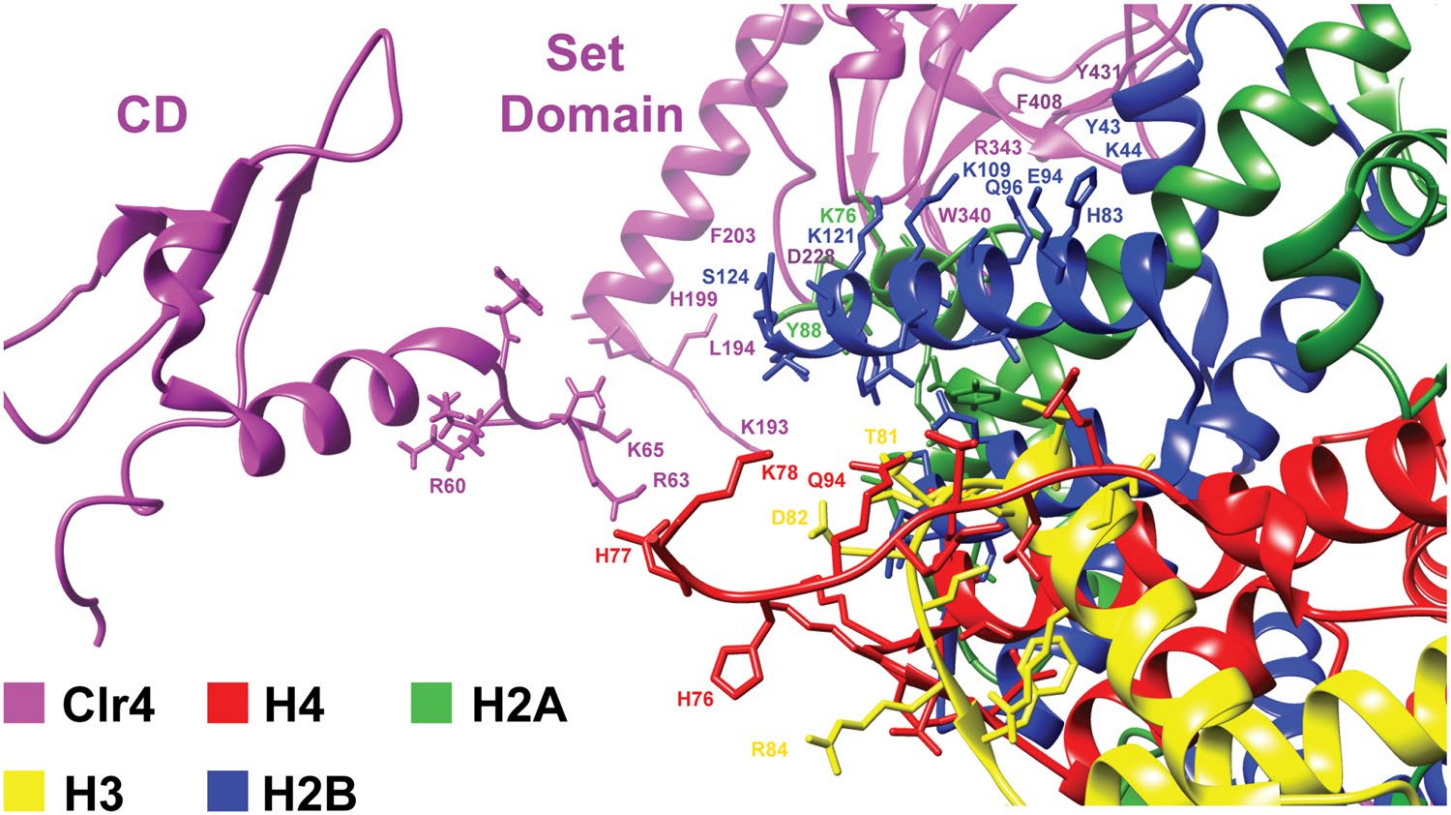
Interaction of Clr4 CD and Set Domain with H3KC9me3 Nucleosome. Key residues important for interaction of Clr4 with the H3KC9me3 nucleosome were identified after molecular docking based on the NMR and cryo-EM results. These included patch ^62^RRLK^65^ with H4 (H77, K78); patch ^190^NPSKL^194^ with H4 (K78, Q94) and H2A (S124); patch ^196^SYT^198^ and ^201^SFY^203^ with H2A (Y88) and H2B (S124); patch ^227^VDDE^230^ with H2A (K76) and H2B (K121); patch ^340^WGVRSLRF^347^ with H2B (E94, Q96, K109); patch ^405^SRFFNH^410^ with H2B (H83); and patch ^431^YDLAFF^436^ with H2B (Y43, K44). Several side chains of the residues are shown in stick representation.

After deciphering the cryo-EM map of free and Clr4-bound H3KC9me3 nucleosome complexes (Fig. 3A,B), we docked the crystal structure of the H3KC9 nucleosome core particle (PDB 1AOI)^1^, the NMR solution structure of Clr4 CD (PDB 1G6Z)^50^ and the crystal structure of Clr4 Pre-SET-post domains (PDB 1MVH)^51^ with the aid of our NMR data (Fig. 5A,B). Docking of the corresponding secondary structures revealed an extra density which explains the interactions of the Clr4 1–191 and Clr4 192–490 constructs with the H3KC9me3 methylated nucleosome core as accessed by NMR spectroscopy. The resulting chemical shifts and intensity ratios are sensitive probes that highlight secondary structures and conformational changes of the Clr4 protein as well as interaction interfaces with its binding partner, the H3KC9me3 nucleosome. Our NMR/cryo-EM data confirms that Clr4 interacts with the nucleosome by binding to patches mediated by a combination of hydrophilic, aromatic and van der Waals contacts. For instance, we have observed the following interactions that constitute the Clr4-H3KC9me3 nucleosome binding interface (Fig. 6): interaction of the arginine anchor in Clr4’s loop patch ^62^RRLK^65^ with histone H4 (H77, K78); loop patch ^190^NPSKL^194^ with histones H4 (K78, Q94) and H2A (S124); the aromatic residues in alpha-helix 4 (^196^SYT^198^ & ^201^SFY^203^) with histones H2A (Y88) and H2B (S124); the acidic loop patch ^227^VDDE^230^ with histones H2A (K76) and H2B (K121); R343 arginine anchor in beta sheet 8 ^340^WGVRSLRF^347^ with histone H2B (E94, Q96, K109); alpha-helix 8 ^405^SRFFNH^410^ with histone H2B (H83) and beta sheet 14 ^431^YDLAFF^436^ with histone H2B (Y43, K44).

**Figure 7:**
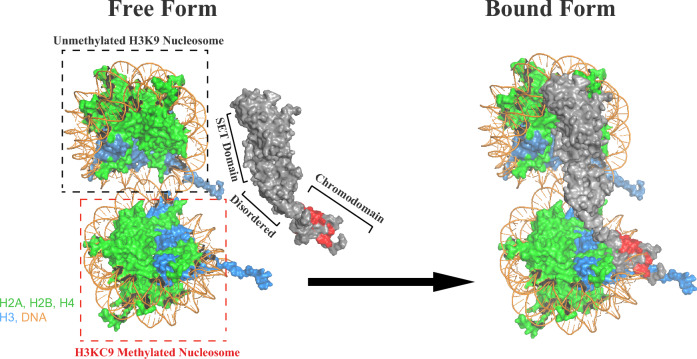
Structural model of The Clr4-H3KC9me3 nucleosome complex. The Cryo-EM/NMR model of Clr4 binding to the H3KC9me3 nucleosome is denoted by space-filling representation. The histone proteins and DNA are color-coded (H2A, H2B, H4 in green, H3 in cyan and DNA in orange) and two nucleosome units are shown: the unmethylated and the H3KC9-methylated, respectively. The domains of full-length Clr4 are indicated. In its free form, the H3KC9-methylated nucleosome is approached by the Clr4 CD (red space-filling representation) to bind the H3 histone protein. In the bound form, the disordered part of Clr4 reinforces this interaction by tethering to both the methylated and unmethylated H3KC9 nucleosomes. This action brings the SET domain closer to the unmethylated H3KC9 nucleosome, for specific methylation of the H3 protein.

Based on the combined experimental outcome, we generated the Cryo-EM/NMR model of Clr4 binding to the H3KC9me3 nucleosome (Fig. 7). In its free form, the H3KC9 methylated nucleosome is approached by the CD of Clr4 to bind the H3 histone protein. Once bound, the disordered part of Clr4 reinforces this interaction by tethering to both the methylated and unmethylated H3KC9 nucleosomes. This action brings the SET domain closer to the unmethylated H3KC9 nucleosome, for specific methylation of the H3 protein. This model is supported by accumulating research^54–56^, where it is now accepted that the first methylated H3KC9 is formed after Clr4 interacts to stabilize the methyltransferase binding to an unmodified nucleosome. Initial CD bindings to the first methylated H3KC9 favors additional H3KC9 methylations.

## Conclusion

Cryo-EM exclusively complements NMR studies of individual proteins or domains and assists in generating mechanistic models to describe the relevant physiological functions. Proteins are inherently dynamic, exhibiting a conformational flexibility that implicates structural rearrangements during molecular interactions and partner binding. Static structural models of proteins therefore display limited insights into the underlying conformational equilibrium. Fortunately, and among all high-resolution techniques, NMR spectroscopy is uniquely adequate for the study of a broad spectrum of these dynamic processes, as well as for resolving the site-specific motions at atomic level over a vast range of time scales in both folded and unfolded proteins. We herein report the interaction and structural impacts of the H3KC9 methyltransferase Clr4 with the nucleosome using NMR spectroscopy and cryo-EM. We show that the chromodomain and the disordered regions of Clr4 bind the nucleosome core to deposit the initial H3KC9 methylation. The SET domain then approaches an unmethylated nucleosome for transient binding. Importantly, the disordered region of Clr4 plays a crucial role in the spread and establishment of heterochromatin. De novo H3KC9 methylation is formed by nonspecific interactions between the nucleosome and the disordered region. The first methylated H3KC9 is formed after this interaction which stabilizes Clr4 on the unmodified nucleosome. Then, the CD binds to the first methylated H3KC9 and recruits additional H3KC9 methylations. The interaction of disordered regions in chromatin complexes and the H3KC9 methyltransferase activity are essential for cancer propagation, thus emphasizing the significance of understanding the mechanisms of de novo deposition of H3KC9 methylation and chromatin modification.

### Supplementary Information

Supplementary Information.

## Data Availability

Data supporting the findings of this study are available in the main manuscript and the supplementary information. ^1^H ^13^C and ^15^N NMR chemical shift assignments data are included in the supplementary information and deposited in the Biological Magnetic Resonance Bank (BMRB; https://www.bmrb.wisc.edu) under accession number 51755. Cryo-EM densities have been deposited in the Electron Microscopy Data Bank (EMDB; https://www.ebi.ac.uk/emdb/) under the accession code EMD-35060. Other data is available upon request.
