# Supplementary material for: Structural insights into the binding mechanism of Clr4 methyltransferase to H3K9 methylated nucleosome
Source: Sci Rep. 2024 Mar 5;14:5438. doi: 10.1038/s41598-024-56248-2 (PMC10914790; doi:10.1038/s41598-024-56248-2)
Supplement: Supplementary file 1 — Supplementary Information. [file 41598_2024_56248_MOESM1_ESM.pdf]

Structural Insights into the Binding Mechanism of Clr4 Methyltransferase to H3K9 Methylated Nucleosome

Christopher Saab[1,2] and Joseph Stephan[3], Elias Akoury[1,*]

[1] Department of Natural Sciences, Lebanese American University, Beirut, 1102-2801, Lebanon
[2] Department of Chemistry, McGill University, 801 Sherbrooke St. West, Montreal, Quebec H3AOB8, Canada
[3] School of Medicine, Lebanese American University, Byblos, PO Box 36, Lebanon

* To whom correspondence should be addressed. Tel: +961 1 786456; Fax: +961 1 786454;
Email: elias.akoury@lau.edu.lb

Supplementary Information

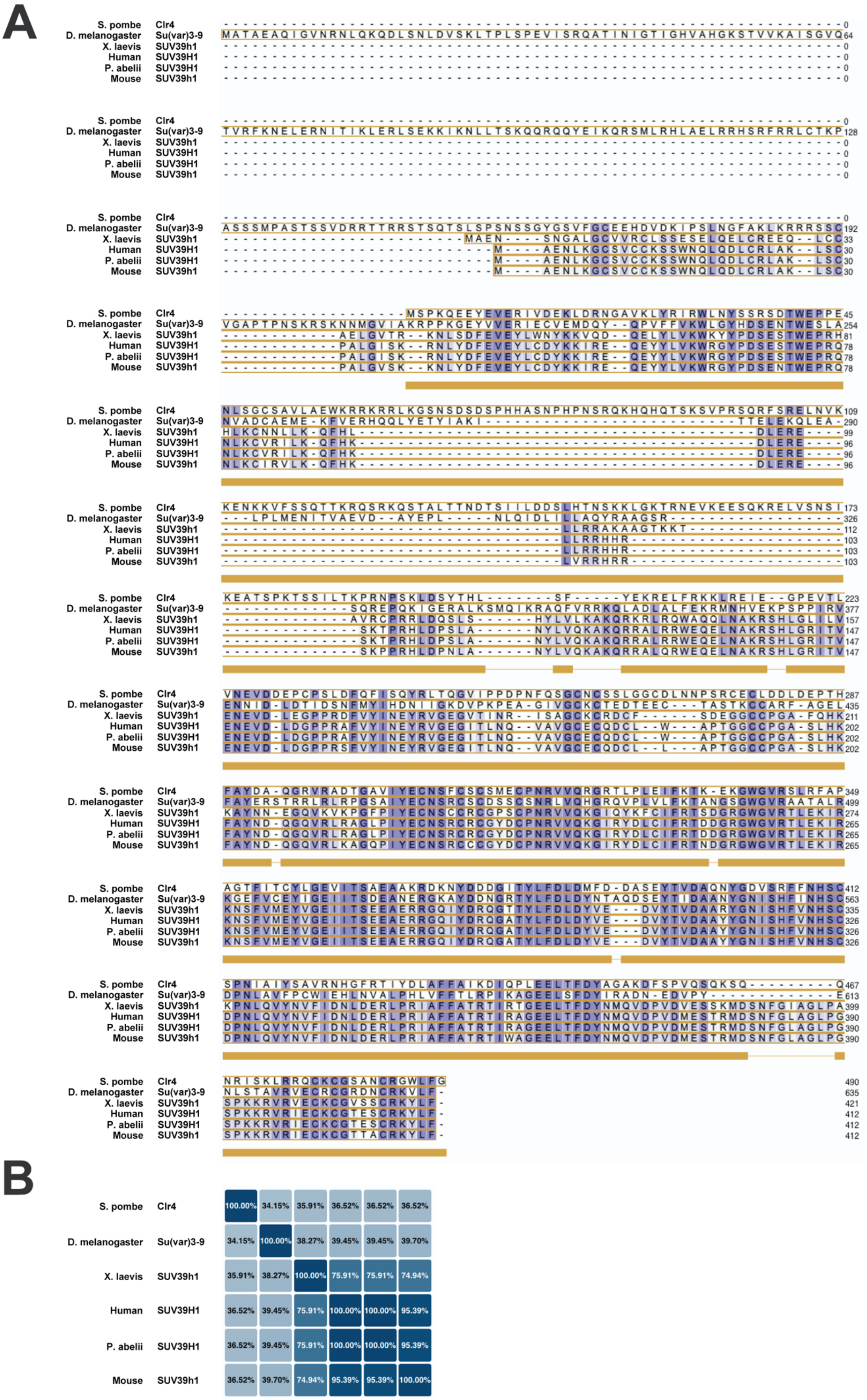
A
S. pombe Clr4
D. melanogaster Su(var)3-9
X. laevis SUV39h1
Human SUV39H1
P. abelii SUV39H1
Mouse SUV39h1
B
S. pombe Clr4 100.00% 34.15% 35.91% 36.52% 36.52% 36.52%
D. melanogaster Su(var)3-9 34.15% 100.00% 38.27% 39.45% 39.45% 39.70%
X. laevis SUV39h1 35.91% 38.27% 100.00% 75.91% 75.91% 74.94%
Human SUV39H1 36.52% 39.45% 75.91% 100.00% 100.00% 95.39%
P. abelii SUV39H1 36.52% 39.45% 75.91% 100.00% 100.00% 95.39%
Mouse SUV39h1 36.52% 39.70% 74.94% 95.39% 95.39% 100.00%

Supplementary Figure 1 – Conserved domains of S. pombe, Drosophila and mammalian Su(var)3-9 proteins. **(A)** Multiple sequence alignment for the Su(var)3-9 family of methyltransferases from different species including Clr4 from Schizosaccharomyces pombe (490 residues), Su(var)3-9 from Drosophila melanogaster (635 residues), SUV39h1 from Xenopus Laevis (421 residues) and mouse (412 residues), SUV39H1 from human (412 residues) and Pongo abelii (412 residues). Conserved domains are highlighted in purple and **(B)** the percent identity matrix reports the sequence similarity that are conserved between the different proteins where Clr4 displays an average of 37% identity with the Su(var)3-9 proteins. Multiple sequence alignment methods are vital for identifying highly conserved residues that are essential for the structure, function, and dynamics of the protein.

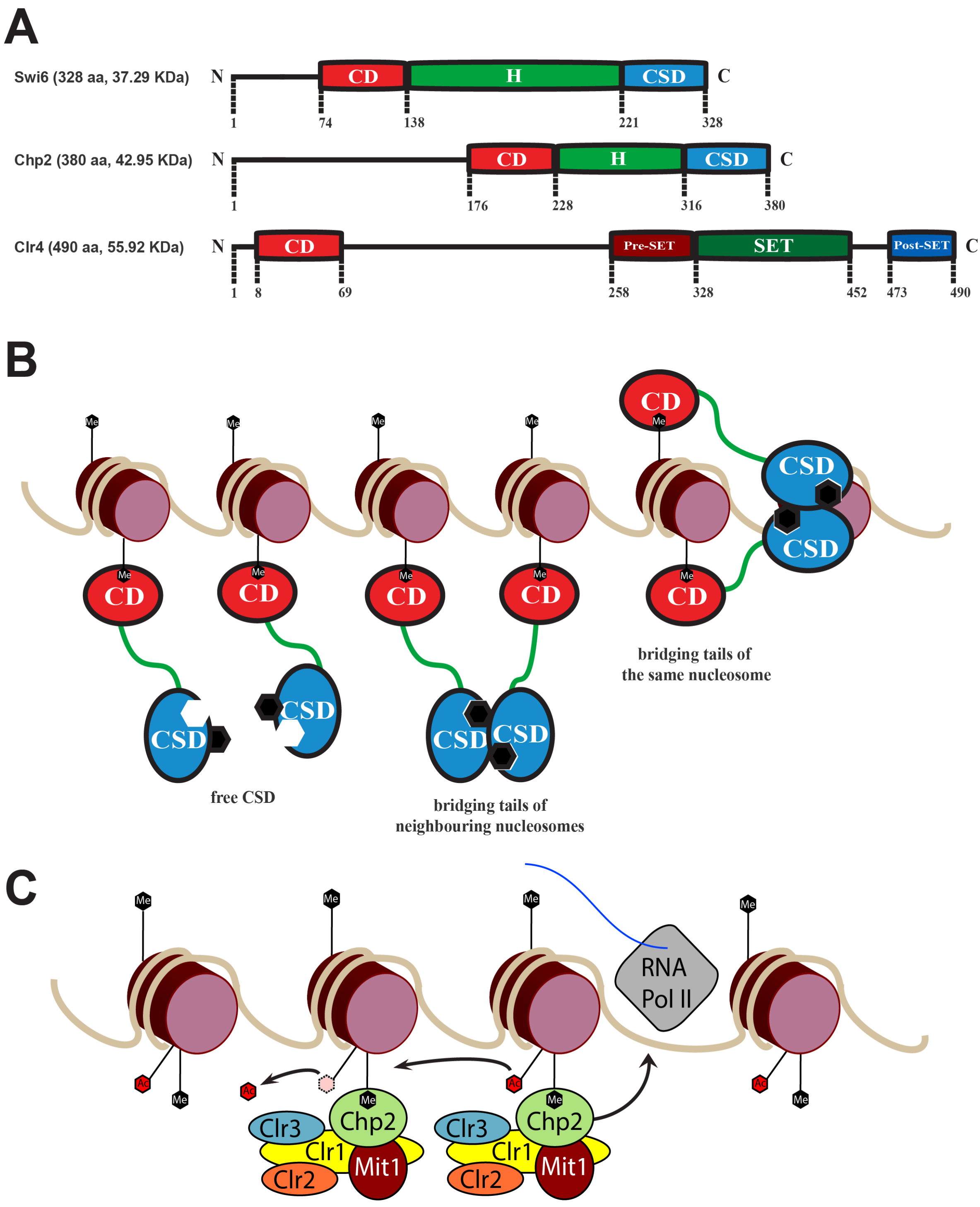

Supplementary Figure 2 – Domain Organization and dimerization of Clr4, Swi6 and Chp2 (A) A schematic diagram representing the domain organization of the full-length Swi6, Chp2 and Clr4 proteins. N = N-terminal, C = C-terminal, CD = Chromodomain, CSD = Chromo shadow domain, SET = SET domain. Schematic drawing of Swi6 and Chp2 depicting the conserved CD and CSD separated by the hinge (H) regions. The numbering is based on the primary sequence of the proteins. **(B)** Swi6 and Chp2 bind H3K9-methylated nucleosomes through the CD and dimerizes through the CSD to bridge H3K9 tails of the same or neighboring nucleosomes. **(C)** A model showing the interaction of Chp2 with SHREC deacetylase complex to form SHREC2. SHREC consists of the four proteins Clr1, Clr2, Clr3 and Mit1. SHREC2 then binds to H3K9me through the Chp2 component, which activates Clr3-mediated deacetylation of H3K14 and the downstream action of Mit1 to limit RNA Pol II access, regulation of nucleosome positioning and processing of the heterochromatin.

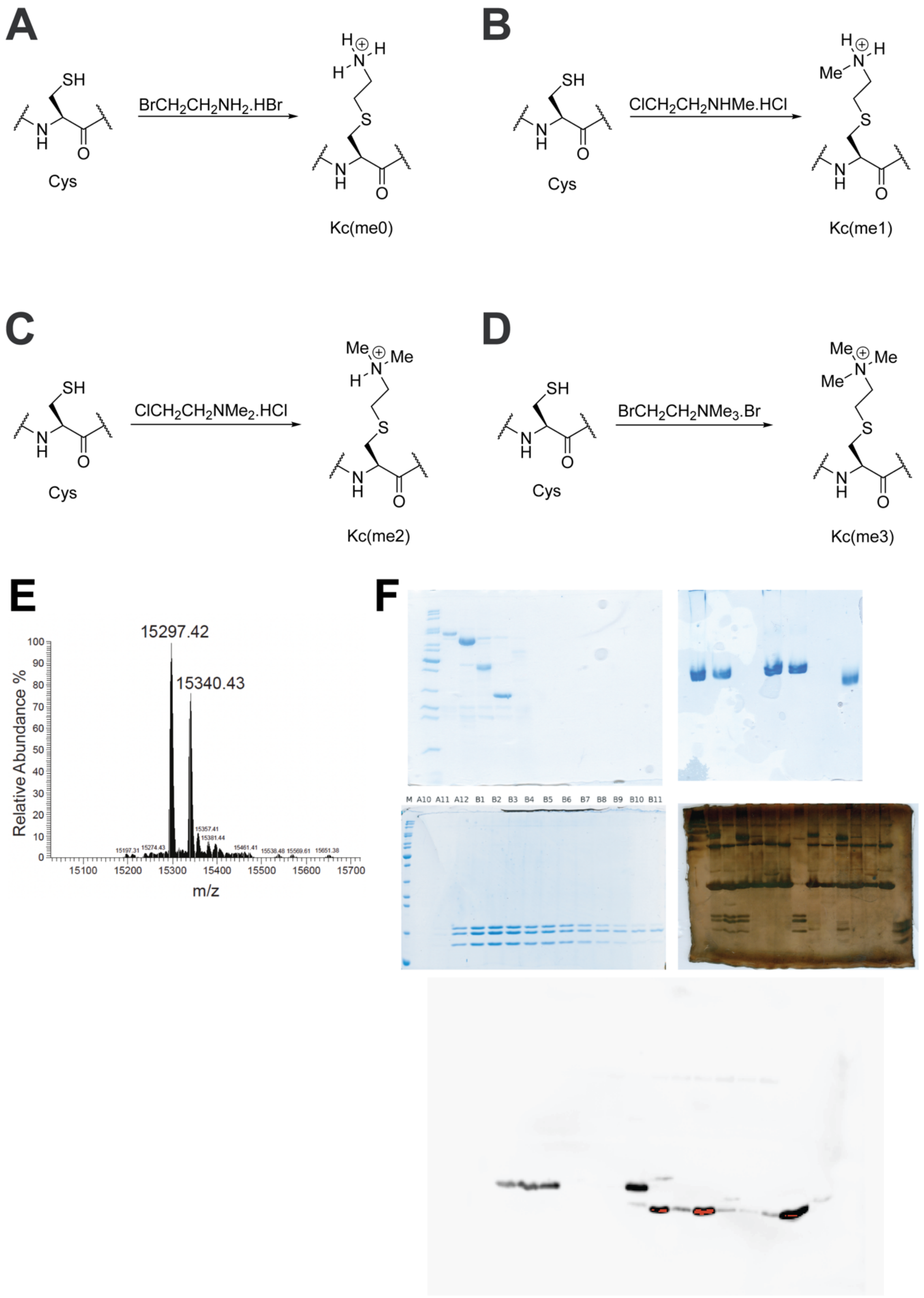

Supplementary Figure 3 – H3KC9me3 Methylation by the Methyl Lysine Analogs Method. Methyl lysine analogs are obtained upon alkylating unique cysteine residues in full-length histones. By using different methylating agents, the analogs can contain **(A)** an unmodified lysine, **(B)** a monomethyl lysine, **(C)** a dimethyl lysine, **(D)** or a trimethyl lysine. **(E)** Mass Spectrometry (MS) analysis of H3KC9me3 histone showing a peak at 15297 Da. **(F)** The full-length membranes of all gels that are reported in figure 2 in the main manuscript; are represented here.

A

Amino Acid	A	C	D	E	F	G	H	I	K	L	M	N	P	Q	R	S	T	V	W	Y
Percentage	4.9	3.2	6.7	6.7	4.9	4.7	2.0	4.1	7.2	7.1	0.6	5.5	4.9	4.4	7.7	10.8	5.5	4.7	1.1	3.3

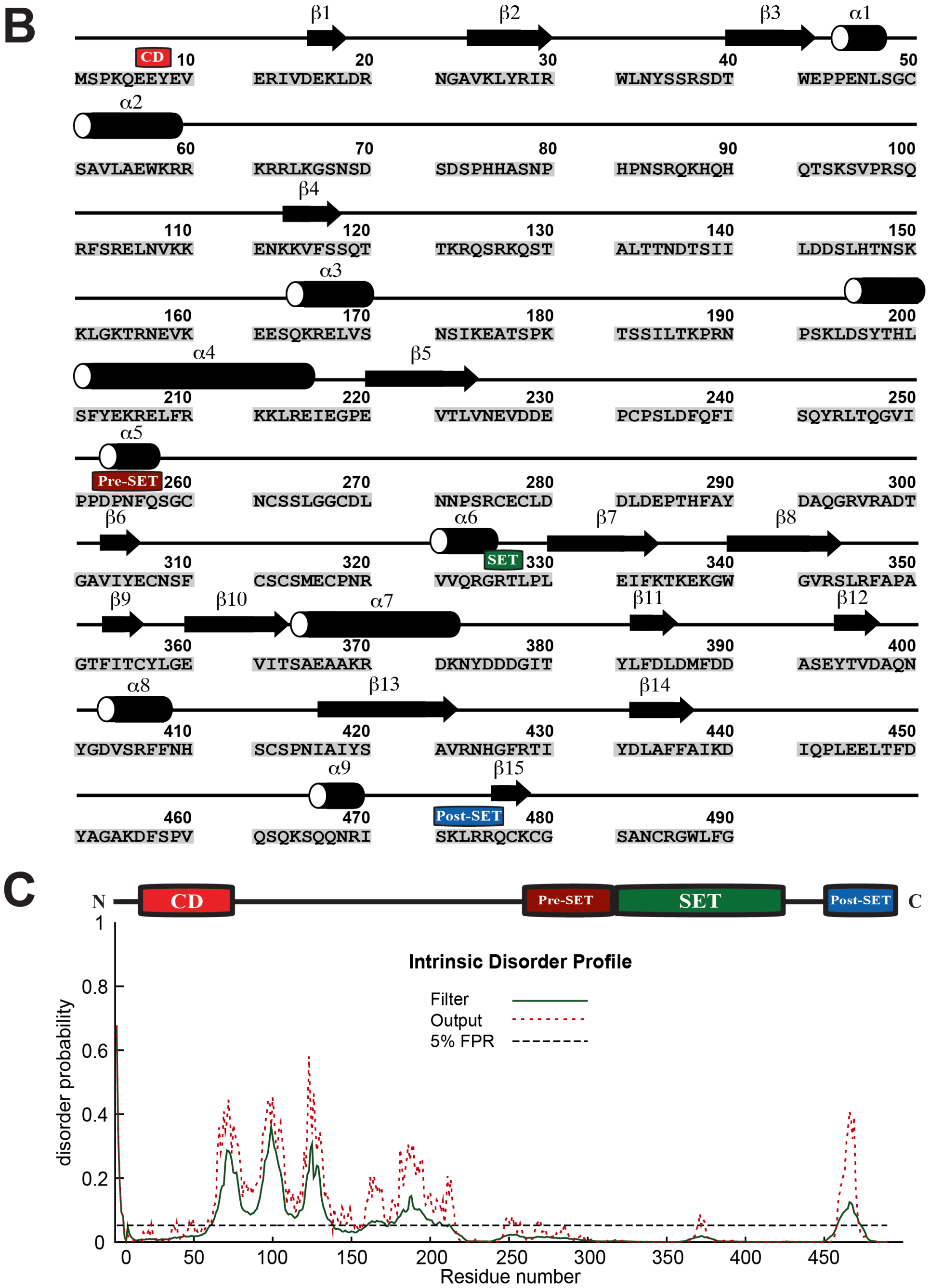

Supplementary Figure 4 – Secondary Structure Analysis of FL Clr4. (A) percentage distribution of amino acids along the Clr4 sequence. **(B)** Primary sequence of full length Clr4 showing the different domains and associated secondary structures. Rods represent alpha helices and arrows represent beta sheets. **(C)** Disorder profile of FL Clr4 showing the disordered patches in the sequence against the probability of disorder; residues 69-191 are classified as disordered at the default threshold.[2]

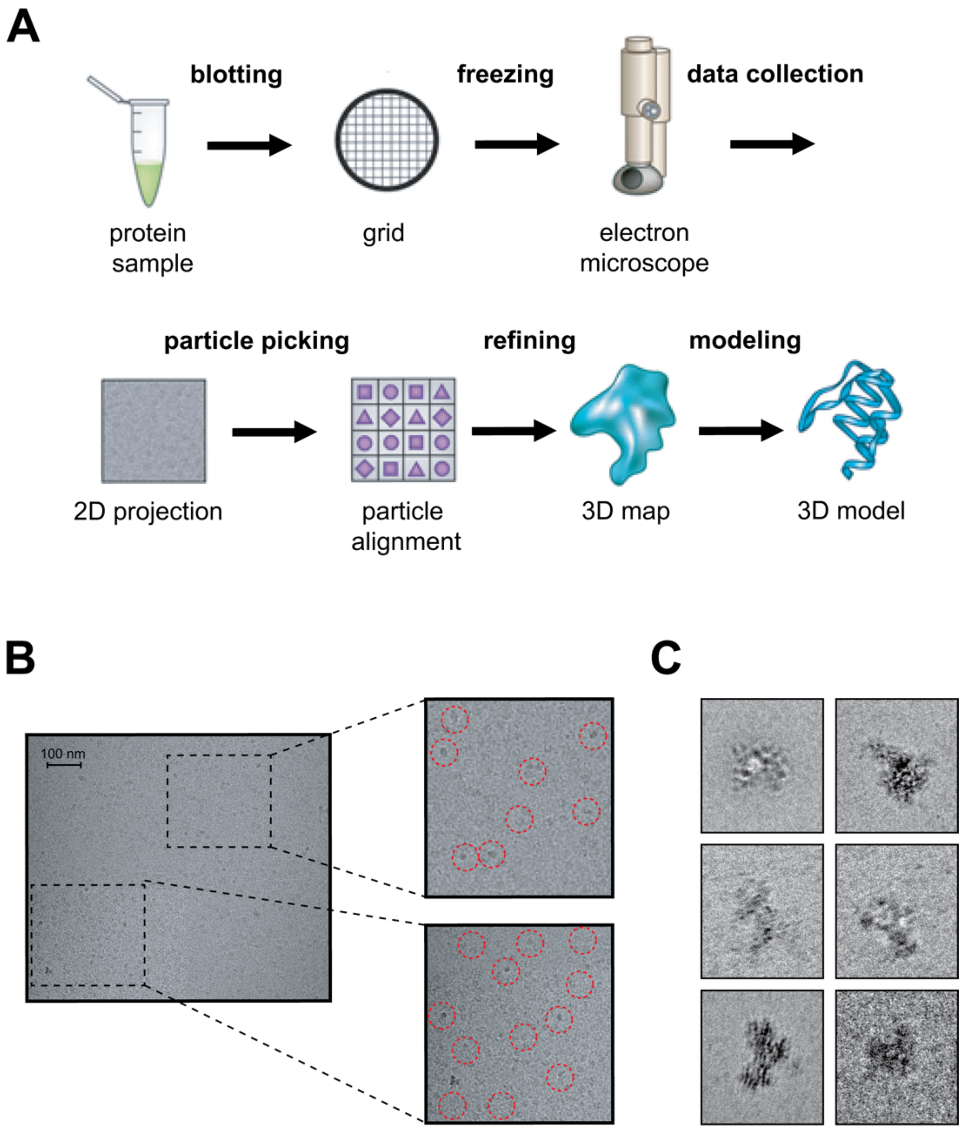

Supplementary Figure 5 – Cryo-electron microscopy workflow: **(A)** a purified protein sample is applied to a copper mesh grid covered with a film of Holey carbon. Upon blotting, it is vitrified with liquid ethane. Particles embedded in the thin ice with various random orientations are then imaged by transmission electron microscopy (TEM). Single particles are selected, aligned for two-dimensional/three-dimensional classification, and iteratively refined to reconstruct a 3D map. Further modeling provides a high-resolution cryo-EM structure. **(B)** Representative cryo-EM micrograph collected with Titan Krios electron microscope at accelerating voltage of 300 kV display highlighted single particles of nucleosomes in different orientations. **(C)** Representative 2D class averages showing Clr4 bound to H3KC9me3 nucleosomes in different orientations.

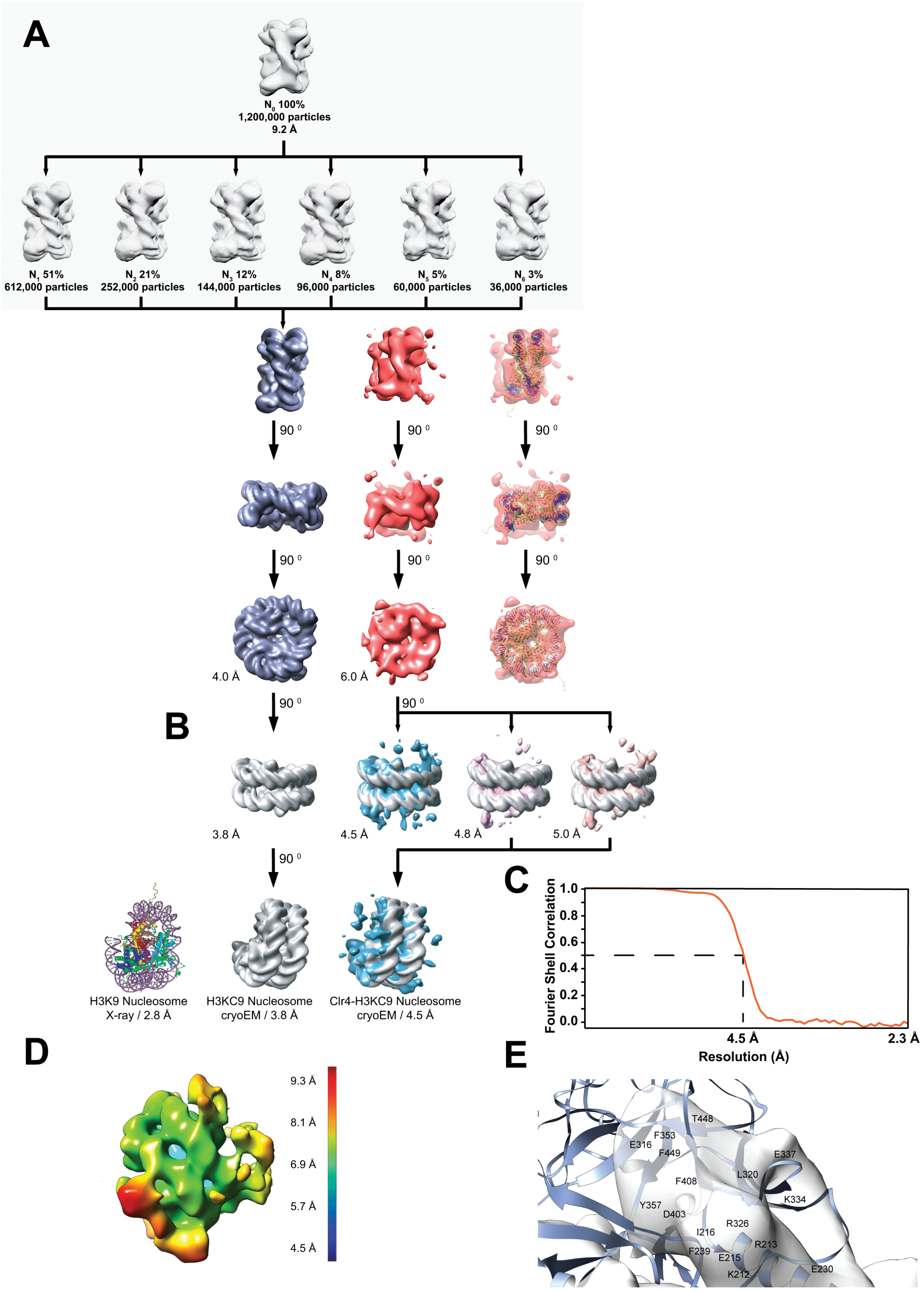
A
N0 100%
1,200,000 particles
9.2 Å
N1 51%
612,000 particles
N2 21%
252,000 particles
N3 12%
144,000 particles
N4 8%
96,000 particles
N5 5%
60,000 particles
N6 3%
36,000 particles
90 °
4.0 Å
6.0 Å
B
3.8 Å
4.5 Å
4.8 Å
5.0 Å
H3K9 Nucleosome
X-ray / 2.8 Å
H3KC9 Nucleosome
cryoEM / 3.8 Å
Clr4-H3KC9 Nucleosome
cryoEM / 4.5 Å
C
Fourier Shell Correlation
1.0
0.8
0.6
0.4
0.2
0.0
4.5 Å
2.3 Å
Resolution (Å)
D
9.3 Å
8.1 Å
6.9 Å
5.7 Å
4.5 Å
E
T448
F353
E316
F449
E337
L320
F408
K334
Y357
D403
R326
I216
R213
F239
E215
E230
K212

Supplementary Figure 6 – Classification of H3KC9me3 Nucleosomes. (A) From a total of 1,200,000 particles, class N_1 contains 51% of all particles and shows the nucleosome core particle. Classes N_2–N_6 show moderate to weak density of the nucleosome core particle. **(B)** The density map of free nucleosome was refined to 3.8 Å (purple) while the density map Clr4-H3KC9 nucleosome complex was refined to 4.5 Å (red). The classification shown on the Clr4-H3KC9me3 complex maps represents the additional densities from Clr4. The x-ray structure of the H3K9 nucleosome core particle (1AOI at 2.8 Å)[3] was docked into the Clr4-H3KC9me3 nucleosome cryo-EM map (rainbow colors). **(C)** Fourier Shell Correlation (FSC) curve showing the resolution of Clr4-H3KC9 Nucleosome Cryo-EM map. The reported resolution is at a cutoff of 0.5. **(D)** Local resolution for Clr4-H3KC9me3 nucleosome Cryo-EM map was calculated with Chimera. **(E)** Ribbon and density diagram (8.0σ, threshold 0.204) of Clr4 binding to H3KC9me3 nucleosome where selected residues from Clr4 set domain (Y, F, E, R, K, and I) are shown.

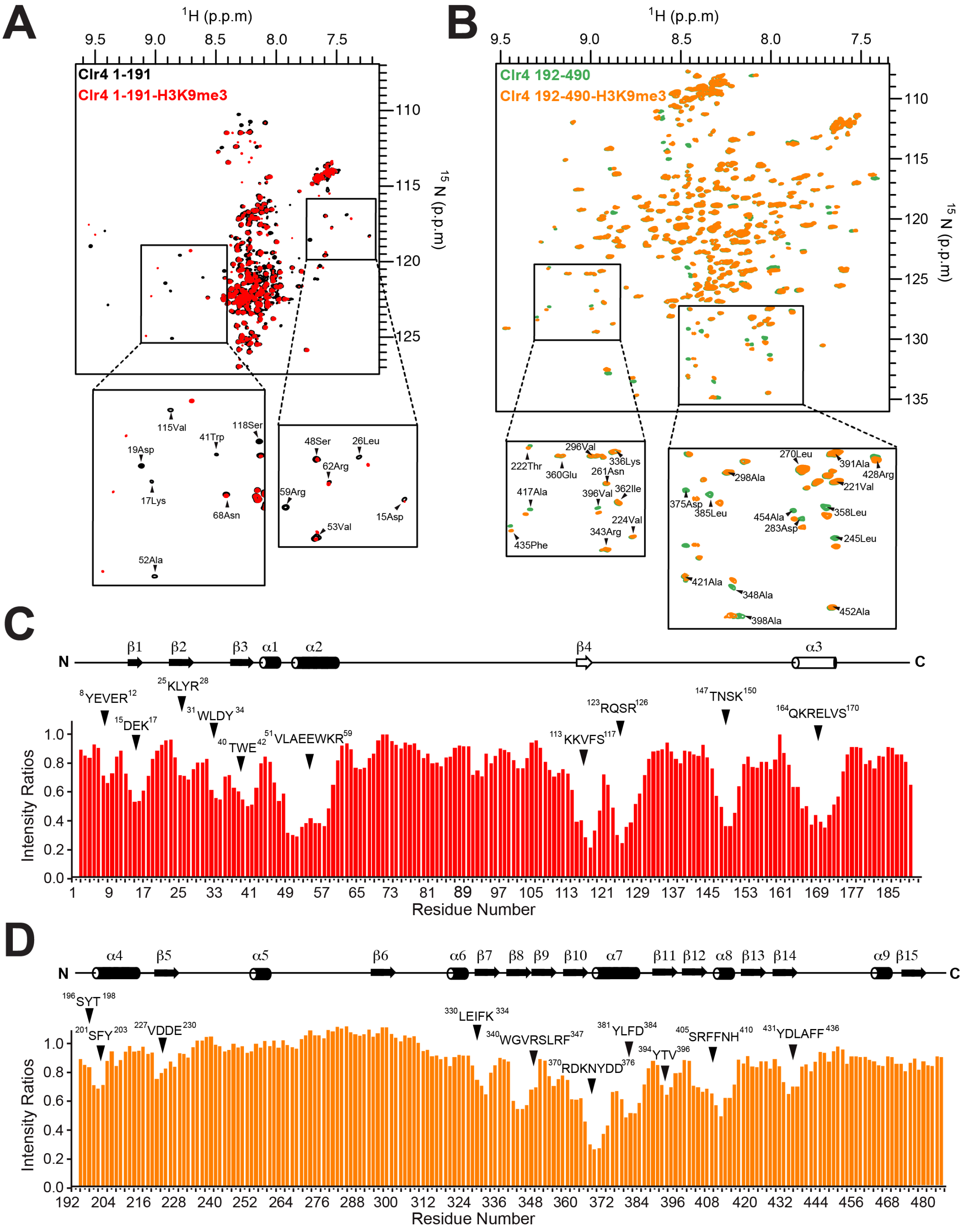

Supplementary Figure 7 – The interaction between Clr4 and H3KC9me3 nucleosome. (**A**) Overlay of two-dimensional ^{1}H-^{15}N HSQC spectra of the isotope-labeled Clr4 1–191 construct in the absence (black) and in presence (red) of unlabeled H3KC9me3 nucleosome (Clr4 1-191:H3KC9me3 nucleosome 1:0.5 ratio). (**B**) Overlay of two-dimensional ^{1}H-^{15}N HSQC spectra of the isotope-labeled Clr4 192–490 construct in the absence (green) and in presence (orange) of unlabeled H3KC9me3 nucleosome (Clr4 192–490:H3KC9me3 nucleosome 1:0.5 ratio). The insets highlight selected chemical shift assignments and the intensity broadening of certain resonances. Intensity ratio plots of (**C**) Clr4 1–191 and (**D**) Clr4 192–490 in the presence of H3KC9me3 nucleosome. Intensity ratios represent the backbone amide resonances with the residue patches of the chromodomain, disordered regions, and Set domain showing the most significant decrease in signal intensities in presence of H3KC9me3 nucleosomes. The secondary structure elements of Clr4 are represented.

Supplementary Table 1. List of Plasmids, oligonucleotides and protein tags used in this study.

List of Plasmids

p406	pET3- Xenopus laevis histone H2A
p407	pET3- Xenopus laevis histone H2B
p408	pET3- Xenopus laevis histone H3
p409	pET3- Xenopus laevis histone H4
P496	pET30a- 6His-3FLAG Clr4 Full-length
p657	pET30a- 6His-3FLAG Clr4 Chromodomain (residues 1-490)
p641	pET30a- 6His-3FLAG Clr4 Delta Chromodomain (residues 70-490)
p645	pET30a- 6His-3FLAG Clr4 PRE-SET-POST (residues 258-490)
p657	pET30a- 6His-3FLAG Clr4 SET-POST (residues 328-490)
p715	pET30a- 6His-3FLAG Clr4 1-191 (residues 1-191)
p717	pET30a- 6His-3FLAG Clr4 192-490 (residues 192-490)
p420	145 bp 601 DNA sequence

List of Primers

470F Clr4 CD	GCAGATTATAAAGATGACGAC
470R Clr4 CD	GTCGGAGTTACTTCCCT
448F Clr4 Delta CD	GACTCGGATTCACCGC
448R Clr4 Delta CD	GGATCCGATATCAGCCATG
449F Clr4 258-590	TCTGGATGCAATTGCTCG
450F Clr4 328-490	CTTCCGCTTGAAATATTTAAAAC
451F Clr4 473-490	CTTCGCCGGCAGTGC
543F Clr4 1-191	GCAGATTATAAAGATGACGACG
543R Clr4 1-191	AGGGTTTCGCGGTTTTG
556F Clr4 192-490	TCCAAACTTGACTCTTATACC
556R Clr4 192-490	CATGGATCCGATATCAGC

Protein Tags

His- & S-tags:	MHHHHHHSSGLVPRGSGMKETAAAKFERQHMDSPDLG TDDDDKAMADIG
Flag-tag:	DYKDDDDK

Supplementary Table 2. Cryo-EM data collection, refinement, and validation statistics

	H3KC9 Nucleosome	Clr4-H3KC9 Nucleosome
**Data collection and processing**		
Magnification	113,000 x	113,000 x
Voltage (kV)	300	300
Electron exposure (e–/Å^2)	100	100
Defocus range (μm)	– 4.0 to – 1.0	– 4.0 to – 1.0
Pixel size (Å^2)	1.2	1.2
Symmetry imposed	C2	C1
Initial particle images (no.)	1,200,000	1,200,000
Final particle images (no.)	612,000	612,000
Map resolution (Å)	3.8	4.5
FSC threshold	0.143	0.143
**Refinement**		
Initial model used	3LZ1	3LZ1
Model resolution (Å)	3.8	4.5
Model resolution range (Å)	235 – 3.8	235 – 4.5
Map sharpening *B* factor (Å^2)	121	100
Model composition		
Non-hydrogen atoms	11 750	12 255
R.m.s. deviations		
Bond lengths (Å)	0.007	0.009
Bond angles (°)	1.134	1.150
Validation		
MolProbity score	1.35	1.42
Clashscore	3.62	11.5
Ramachandran plot		
Favored (%)	98.2	95
Allowed (%)	1.8	5
Disallowed (%)	0.0	0
**EMDB**	-	35060

Supplementary Table 3. Chemical Shift Assignments of Full length Clr4

Residue	δ_{HN}	$\delta_{C\alpha}$	$\delta_{C\beta}$	δ_{N}	$\delta_{C'}$
**4 Lys**	8.33	56.57	33.45	120.78	176.42
**5 Gln**	8.37	56.33	29.31	121.91	175.54
**7 Glu**	8.32	54.84	30.29	120.95	175.84
**8 Tyr**	8.18	62.61	38.53	121.39	175.19
**9 Glu**	8.26	53.14	29.16	121.32	175.84
**10 Val**	8.36	62.07	30.74	115.79	175.46
**11 Glu**	8.27	55.44	31.46	117.65	174.05
**12 Arg**	8.52	58.53	31.14	117.83	174.45
**13 Ile**	8.16	61.56	35.43	123.04	175.06
**14 Val**	8.17	62.29	33.44	124.32	175.11
**15 Asp**	6.96	54.04	44.43	117.71	174.51
**16 Glu**	8.86	54.52	33.61	118.52	174.24
**17 Lys**	8.89	55.41	34.97	122.36	175.58
**18 Leu**	8.57	54.06	43.14	125.16	176.71
**19 Asp**	8.35	53.06	42.94	118.69	177.32
**20 Arg**	8.46	57.88	29.59	117.11	176.79
**21 Asn**	8.37	53.27	39.05	117.15	175.88
**22 Gly**	8.12	44.10	–	108.75	174.51
**23 Ala**	8.28	50.95	19.65	123.48	177.61
**24 Val**	7.72	60.65	32.31	115.71	176.01

**25 Lys**	8.16	57.09	34.48	117.29	175.02
**26 Leu**	7.79	53.66	46.83	118.97	175.09
**27 Tyr**	9.09	56.73	43.21	117.16	174.03
**28 Arg**	8.73	54.19	32.19	122.89	174.02
**29 Ile**	8.05	59.92	39.44	126.54	174.12
**30 Arg**	8.08	55.93	31.53	124.03	175.21
**31 Trp**	8.29	57.08	30.57	121.91	175.12
**32 Leu**	8.31	56.79	41.97	126.05	178.71
**33 Asn**	8.85	55.86	38.24	118.19	176.48
**34 Tyr**	8.33	56.64	38.12	118.51	176.69
**35 Ser**	7.89	58.67	63.79	115.18	175.29
**36 Ser**	8.23	56.34	62.34	116.77	173.63
**37 Arg**	7.76	56.15	31.92	119.56	174.69
**38 Ser**	8.91	59.01	63.71	118.41	173.19
**39 Asp**	8.61	52.62	42.99	121.67	174.94
**40 Thr**	8.64	60.93	71.88	112.14	172.51
**41 Trp**	8.83	56.26	31.58	121.44	175.98
**42 Glu**	9.63	55.82	33.85	122.45	173.77
**45 Glu**	9.19	59.41	28.88	116.19	178.35
**46 Asn**	7.68	54.45	38.62	115.16	176.19
**47 Leu**	8.18	54.87	41.03	118.67	178.22
**48 Ser**	7.46	59.16	62.81	112.76	174.84

**49 Gly**	8.95	43.59	–	111.22	173.93
**50 Cys**	8.18	58.35	26.65	118.56	174.93
**51 Ser**	8.12	61.82	63.11	116.15	176.43
**52 Ala**	8.25	55.08	18.49	123.08	179.26
**53 Val**	7.21	66.31	31.57	118.34	177.79
**54 Leu**	7.99	61.31	41.35	122.51	178.89
**55 Ala**	7.75	54.97	18.31	120.91	180.02
**56 Glu**	8.04	59.17	29.75	119.23	178.57
**57 Trp**	8.03	60.46	30.17	122.31	177.80
**58 Lys**	8.52	59.31	32.31	118.59	178.97
**59 Arg**	7.81	58.24	30.08	118.38	178.05
**60 Arg**	7.54	57.35	30.97	116.96	177.28
**61 Lys**	7.86	59.39	32.49	119.21	177.53
**62 Arg**	7.56	57.88	30.15	119.10	178.22
**63 Arg**	7.55	57.87	30.28	120.19	176.62
**64 Leu**	8.03	55.64	41.59	122.36	177.08
**65 Lys**	8.82	57.31	31.52	125.54	176.27
**66 Gly**	8.49	45.46	–	108.49	173.92
**67 Ser**	8.03	59.21	64.72	118.49	173.61
**68 Asn**	8.46	54.61	39.89	118.13	174.82
**69 Ser**	8.19	58.33	63.86	118.76	174.36
**70 Asp**	8.44	54.62	40.88	122.53	176.44

**71 Ser**	8.14	56.60	63.73	116.88	174.59
**72 Asp**	8.50	56.06	63.71	121.93	176.22
**73 Ser**	8.14	57.21	63.81	117.02	174.26
**77 Ala**	8.49	50.77	19.03	124.70	177.75
**78 Ser**	8.21	57.34	63.47	113.69	174.87
**79 Asn**	8.42	53.58	38.71	119.51	175.23
**83 Asn**	8.13	53.74	38.52	118.09	175.41
**84 Ser**	8.42	56.47	63.14	118.52	174.24
**85 Arg**	8.32	56.57	30.68	122.93	176.43
**86 Gln**	8.34	54.72	29.18	120.25	176.35
**87 Lys**	8.48	56.36	32.78	123.50	176.64
**89 Gln**	8.33	54.68	29.19	120.53	176.14
**91 Gln**	8.28	55.12	29.64	121.05	176.38
**92 Thr**	8.12	61.85	69.59	115.71	174.46
**93 Ser**	8.41	59.81	63.78	116.25	174.22
**94 Lys**	8.42	56.60	32.74	125.18	176.84
**95 Ser**	8.33	56.74	63.74	117.44	174.87
**96 Val**	8.19	62.30	32.71	116.86	175.65
**98 Arg**	8.46	56.59	30.72	121.77	176.35
**99 Ser**	8.30	58.79	63.78	115.44	175.59
**100 Gln**	8.55	56.56	29.11	120.37	176.97
**101 Arg**	8.53	56.09	31.05	121.75	176.84

**102 Phe**	8.29	58.82	39.92	120.16	175.48
**103 Ser**	8.05	58.22	63.47	118.14	175.88
**104 Arg**	8.40	56.54	30.93	122.93	176.24
**105 Glu**	8.52	56.58	30.15	121.77	176.85
**106 Leu**	8.26	56.82	42.26	122.12	177.04
**107 Asn**	8.41	53.58	38.72	116.36	176.21
**108 Val**	8.28	62.08	32.87	120.27	175.65
**109 Lys**	8.36	56.59	32.89	122.93	176.98
**110 Lys**	8.45	58.33	33.05	123.57	177.14
**111 Glu**	8.26	56.85	30.25	121.58	177.21
**112 Asn**	7.83	53.21	38.92	119.42	175.31
**113 Lys**	8.36	56.60	32.54	122.93	176.57
**114 Lys**	8.71	56.95	32.78	122.41	176.64
**115 Val**	8.96	63.63	32.87	119.15	176.24
**116 Phe**	8.42	57.63	39.74	125.75	175.49
**117 Ser**	8.04	54.48	63.77	117.92	174.32
**118 Ser**	8.46	58.58	63.54	117.78	174.65
**119 Gln**	8.42	54.34	29.74	120.35	176.44
**120 Thr**	8.21	61.84	69.62	115.92	176.75
**121 Thr**	8.24	62.33	69.24	115.02	176.28
**122 Lys**	8.33	55.26	32.64	125.75	176.24
**123 Arg**	8.46	56.61	30.62	122.47	176.41

**124 Gln**	8.26	56.36	29.98	120.84	176.22
**125 Ser**	8.38	58.69	63.74	118.26	174.59
**126 Arg**	8.42	56.57	30.44	122.93	176.17
**127 Lys**	8.42	56.60	32.17	122.29	176.41
**128 Gln**	8.35	56.47	29.77	122.08	176.33
**129 Ser**	8.31	58.69	63.72	117.99	174.68
**130 Thr**	8.19	62.09	69.59	115.60	174.64
**131 Ala**	8.33	52.62	19.15	126.45	177.74
**132 Leu**	8.53	56.94	43.21	121.97	177.61
**133 Thr**	8.17	61.82	69.82	113.25	174.87
**134 Thr**	8.20	61.87	69.95	115.00	174.96
**135 Asn**	8.35	55.56	38.74	119.73	175.26
**136 Asp**	8.31	54.32	40.88	121.34	176.43
**137 Thr**	8.42	55.30	69.74	109.57	174.84
**138 Ser**	8.33	59.01	63.92	116.39	175.02
**139 Ile**	8.10	62.09	38.56	121.61	175.83
**140 Ile**	8.28	61.39	38.87	126.29	175.92
**141 Leu**	8.38	56.67	42.41	124.06	177.14
**142 Asp**	8.31	55.39	40.58	123.54	176.58
**143 Asp**	8.15	53.17	40.87	123.01	177.02
**144 Ser**	8.21	58.99	63.77	117.49	174.58
**145 Leu**	8.36	56.56	42.74	125.75	177.24

**147 Thr**	8.12	62.14	69.74	115.03	174.74
**148 Asn**	8.45	54.55	38.17	119.49	175.28
**149 Ser**	8.45	58.57	63.98	118.59	174.58
**150 Lys**	8.41	56.58	32.98	125.18	176.64
**151 Lys**	8.45	58.34	32.47	123.50	176.24
**152 Leu**	8.37	55.97	42.54	123.51	177.08
**153 Gly**	8.41	45.32	–	109.79	173.85
**154 Lys**	8.34	56.35	32.72	122.34	176.47
**155 Thr**	8.23	61.62	69.59	114.64	174.65
**156 Arg**	8.52	56.60	30.77	122.93	176.68
**157 Asn**	8.33	52.81	38.25	119.27	175.21
**158 Glu**	8.75	57.02	30.05	120.52	177.02
**159 Val**	8.46	62.42	32.14	121.94	175.74
**160 Lys**	8.54	57.85	32.79	122.46	176.47
**161 Glu**	8.30	55.32	30.58	121.41	176.68
**162 Glu**	8.38	54.57	30.47	121.22	176.84
**163 Ser**	8.37	58.7	64.02	115.44	175.21
**164 Gln**	8.32	56.65	29.41	120.77	176.97
**165 Lys**	7.80	58.09	32.87	120.24	176.87
**166 Arg**	8.62	56.74	30.98	121.14	176.47
**167 Glu**	8.57	57.43	30.17	121.85	176.89
**168 Leu**	8.24	55.87	42.74	122.83	177.24

**169 Val**	8.26	63.32	32.41	121.09	176.12
**170 Ser**	8.34	59.12	63.42	117.18	175.12
**171 Asn**	8.44	56.54	39.12	119.48	175.24
**172 Ser**	8.43	59.07	64.02	118.66	175.35
**173 Ile**	8.17	61.58	38.75	122.35	175.92
**174 Lys**	8.33	57.16	32.84	123.75	176.63
**175 Glu**	8.29	57.36	30.01	121.93	176.74
**176 Ala**	8.45	55.00	19.04	127.66	177.79
**177 Thr**	8.42	62.21	69.59	114.66	175.24
**178 Ser**	8.38	59.37	63.47	116.30	174.84
**180 Lys**	8.43	56.98	33.04	121.53	176.57
**181 Thr**	8.23	62.22	64.87	114.69	174.48
**182 Ser**	8.29	59.12	63. 72	116.17	174.75
**183 Ser**	8.41	59.16	62.87	116.79	175.44
**184 Ile**	8.17	61.52	38.74	122.35	176.21
**185 Leu**	8.40	56.56	42.81	123.50	177.37
**186 Thr**	8.17	61.82	69.41	113.47	174.47
**187 Lys**	8.27	58.87	33.14	126.41	176.97
**189 Arg**	8.44	56.74	30.54	121.65	176.47
**190 Asn**	8.22	55.64	38.67	119.40	175.36
**193 Lys**	8.22	56.23	32.76	120.02	175.19
**194 Leu**	7.74	54.76	42.44	120.13	176.90

**195 Asp**	8.42	52.90	42.07	121.96	176.38
**196 Ser**	8.80	61.65	62.81	116.01	176.28
**197 Tyr**	8.81	61.60	38.03	121.53	178.64
**198 Thr**	8.46	66.69	68.49	117.06	175.86
**199 His**	8.29	59.95	29.67	119.81	177.81
**200 Leu**	8.03	57.36	41.30	119.72	179.15
**201 Ser**	8.37	61.45	62.56	115.73	177.34
**202 Phe**	8.79	60.65	38.20	120.90	177.93
**203 Tyr**	8.19	61.87	38.35	120.81	178.42
**204 Glu**	8.80	59.26	29.66	118.79	179.34
**205 Lys**	8.18	59.41	32.67	120.85	178.62
**206 Arg**	8.20	59.10	29.90	119.59	178.70
**207 Glu**	7.68	58.34	29.10	119.14	179.26
**208 Leu**	7.93	57.80	41.75	120.01	179.45
**209 Phe**	7.89	61.86	39.39	121.48	178.04
**210 Arg**	7.89	58.61	29.82	119.16	178.77
**211 Lys**	7.66	58.70	32.01	120.27	178.36
**212 Lys**	7.80	59.25	32.55	119.96	178.52
**213 Leu**	7.69	56.69	40.96	118.25	179.98
**214 Arg**	7.95	58.17	30.11	118.97	177.48
**215 Glu**	7.44	56.43	29.91	116.21	176.27
**216 Ile**	7.09	61.52	38.34	121.07	175.70
**217 Glu**	8.54	56.56	29.27	127.09	175.96

**218 Gly**	8.13	44.39	–	113.58	171.51
**220 Glu**	8.52	57.68	31.13	120.65	175.63
**221 Val**	7.98	61.24	34.09	124.47	175.14
**222 Thr**	9.24	60.42	71.83	119.57	172.55
**223 Leu**	8.30	53.87	44.70	122.36	175.20
**224 Val**	9.08	60.56	35.67	123.78	174.30
**225 Asn**	8.59	52.83	39.97	123.42	174.37
**226 Glu**	9.04	55.83	30.60	125.04	176.28
**227 Val**	8.11	64.02	32.85	119.09	175.95
**228 Asp**	8.68	53.15	42.05	117.71	175.65
**229 Asp**	8.23	53.83	40.67	118.46	175.88
**230 Glu**	8.68	55.41	30.17	122.24	176.02
**232 Cys**	7.72	56.53	28.85	110.44	171.47
**234 Ser**	8.05	58.96	63.78	115.34	175.59
**235 Leu**	8.49	55.01	42.45	126.37	177.14
**236 Asp**	8.15	54.48	39.86	119.86	175.32
**237 Phe**	7.10	56.57	41.34	115.95	172.23
**238 Gln**	8.46	53.44	32.92	118.27	174.09
**239 Phe**	8.46	58.20	39.91	122.78	175.88
**240 Ile**	8.66	59.57	41.89	117.95	175.01
**241 Ser**	8.64	57.68	64.19	114.67	173.31
**242 Gln**	7.62	54.19	32.44	119.48	174.24
**243 Tyr**	8.34	58.76	38.45	122.02	175.72

**244 Arg**	8.92	54.69	31.13	124.21	175.10
**245 Leu**	8.26	54.93	42.46	126.99	176.76
**246 Thr**	7.93	59.62	71.51	114.51	173.80
**247 Gln**	8.43	57.63	28.46	118.41	176.84
**248 Gly**	8.75	44.56	–	112.53	174.28
**249 Val**	7.34	62.22	31.77	120.46	175.47
**250 Ile**	8.66	58.40	37.93	124.83	174.16
**253 Asp**	8.41	52.68	40.75	126.64	175.98
**255 Asn**	8.84	55.06	37.94	115.95	174.80
**256 Phe**	7.52	57.72	38.45	118.09	175.27
**257 Gln**	7.43	54.66	31.94	118.51	175.86
**258 Ser**	8.71	57.80	63.60	118.18	174.20
**259 Gly**	8.41	44.73	–	112.06	173.41
**260 Cys**	9.03	58.82	28.22	123.11	175.12
**261 Asn**	9.07	52.38	38.86	121.75	175.33
**262 Cys**	8.35	57.97	27.14	116.80	174.04
**263 Ser**	8.07	57.76	62.78	115.73	174.35
**264 Ser**	8.76	59.84	63.17	122.33	174.46
**265 Leu**	8.20	56.04	43.11	122.19	177.61
**266 Gly**	8.30	46.11	–	108.17	174.96
**267 Gly**	8.44	45.52	–	111.37	174.58
**268 Cys**	8.30	58.00	28.19	120.14	173.13
**269 Asp**	7.79	55.00	39.57	118.07	176.25

**270 Leu**	8.30	56.07	41.41	124.24	176.73
**271 Asn**	8.19	52.86	38.54	115.31	174.79
**272 Asn**	7.76	50.90	38.72	117.28	174.57
**274 Ser**	8.47	58.20	63.16	110.59	174.46
**275 Arg**	7.95	56.23	32.21	119.61	176.34
**276 Cys**	7.85	58.75	28.55	118.21	174.53
**277 Glu**	8.86	56.51	29.93	122.38	176.71
**278 Cys**	7.96	58.69	28.22	113.82	173.86
**279 Leu**	7.36	53.85	42.43	116.93	176.63
**280 Asp**	7.49	56.08	40.35	118.80	176.31
**281 Asp**	7.14	54.01	39.95	115.82	175.69
**282 Leu**	7.31	55.92	42.32	120.81	175.12
**283 Asp**	8.38	53.39	41.34	127.54	175.15
**284 Glu**	8.76	56.92	31.34	125.84	173.92
**286 Thr**	7.94	62.65	69.63	118.59	174.20
**287 His**	8.33	55.77	30.92	119.81	173.01
**288 Phe**	8.57	58.12	39.86	120.34	175.76
**289 Ala**	8.62	54.59	18.14	124.01	175.63
**290 Tyr**	7.20	56.11	41.09	113.49	175.38
**291 Asp**	8.36	53.36	41.27	121.23	176.37
**292 Ala**	8.60	54.39	18.66	122.72	178.79
**293 Gln**	8.09	56.34	28.78	115.98	176.78
**294 Gly**	7.61	45.27	–	107.06	174.46

**295 Arg**	7.48	55.81	31.49	119.61	175.72
**296 Val**	8.94	61.99	31.85	120.40	176.08
**297 Arg**	8.42	56.62	30.73	126.65	177.37
**298 Ala**	8.44	53.13	18.50	123.99	177.56
**299 Asp**	8.16	53.36	39.76	116.20	175.93
**300 Tyr**	7.36	61.88	69.87	117.38	174.93
**301 Gly**	8.45	44.99	–	113.94	174.31
**302 Ala**	8.32	52.55	18.05	121.21	175.48
**303 Val**	7.38	61.11	34.43	117.22	174.36
**304 Ile**	8.29	60.12	40.17	127.90	173.84
**305 Tyr**	8.47	56.48	39.38	126.67	175.32
**306 Glu**	8.48	55.64	31.53	123.46	175.90
**307 Cys**	8.17	59.86	28.36	118.49	174.81
**308 Asn**	8.46	52.56	39.36	120.56	176.92
**309 Ser**	8.19	59.66	62.47	112.71	174.96
**310 Phe**	7.97	56.74	38.86	119.50	175.61
**311 Cys**	7.49	57.98	28.89	116.95	174.58
**312 Ser**	8.67	58.06	63.43	114.60	174.45
**313 Cys**	7.79	57.21	29.18	116.85	174.35
**314 Ser**	8.28	57.39	64.33	115.56	176.19
**315 Met**	8.89	56.71	31.69	119.03	176.92
**316 Glu**	7.92	55.70	29.28	117.48	176.19
**317 Cys**	7.42	56.99	27.17	120.20	174.09

**319 Asn**	8.41	52.29	39.21	116.61	175.04
**320 Arg**	7.92	54.79	30.88	118.54	175.90
**321 Val**	7.54	66.24	30.85	120.76	177.63
**322 Val**	8.08	65.76	31.16	118.31	177.66
**323 Gln**	7.73	58.65	28.69	118.48	178.09
**324 Arg**	7.51	56.68	30.42	116.13	176.38
**325 Gly**	7.76	44.77	–	107.71	173.92
**326 Arg**	8.44	57.35	31.02	120.26	176.14
**327 Thr**	8.59	61.79	71.11	116.52	173.75
**328 Leu**	7.50	52.10	42.86	122.50	175.04
**330 Leu**	8.72	53.42	46.27	121.80	175.23
**331 Glu**	8.60	54.69	33.95	121.16	174.89
**332 Ile**	9.31	61.13	38.96	127.77	175.17
**333 Phe**	9.01	55.51	42.59	123.53	172.91
**334 Lys**	8.61	54.90	32.10	122.62	176.37
**335 Thr**	7.90	60.24	70.64	117.03	174.69
**336 Lys**	8.89	59.03	32.60	120.49	177.04
**337 Glu**	8.70	57.55	30.78	116.51	176.90
**338 Lys**	7.36	55.36	33.50	118.78	176.21
**339 Gly**	8.44	46.65	–	107.07	172.83
**340 Trp**	8.56	57.19	30.88	121.84	176.13
**341 Gly**	8.78	44.54	–	107.55	172.35
**342 Val**	8.80	60.69	36.19	118.40	174.32

**343 Arg**	9.26	53.96	33.75	124.71	174.32
**344 Ser**	8.53	59.07	64.51	115.73	175.03
**345 Leu**	8.27	54.54	43.42	124.06	176.55
**346 Arg**	7.72	54.18	33.96	117.77	175.16
**347 Phe**	8.67	58.26	39.56	121.46	174.90
**348 Ala**	8.67	49.38	20.99	128.67	174.36
**350 Ala**	8.68	53.73	18.79	125.20	178.00
**351 Gly**	9.25	45.25	–	110.49	174.35
**352 Thr**	8.02	62.97	69.54	118.71	174.17
**353 Phe**	8.43	57.74	40.31	125.80	173.93
**354 Ile**	7.81	61.69	39.74	124.94	174.62
**355 Thr**	6.85	62.45	71.24	113.09	171.41
**356 Cys**	8.55	57.80	29.65	117.66	173.49
**357 Tyr**	8.80	57.00	38.92	122.36	174.68
**358 Leu**	8.04	53.29	43.20	125.95	176.54
**359 Gly**	8.23	45.69	–	106.70	171.24
**360 Glu**	9.06	56.22	31.34	120.65	175.61
**361 Val**	8.56	62.06	31.50	125.13	175.20
**362 Ile**	9.15	58.62	41.36	122.98	174.97
**363 Thr**	8.46	60.91	70.51	113.40	175.63
**364 Ser**	9.39	61.08	62.69	118.62	175.39
**365 Ala**	8.31	54.26	18.34	123.22	179.27
**366 Glu**	7.93	59.61	29.53	119.56	178.38

**367 Ala**	8.51	55.41	18.46	121.25	179.43
**368 Ala**	8.23	54.89	18.21	120.44	180.32
**369 Lys**	7.75	59.42	32.28	119.08	180.01
**370 Arg**	8.58	59.08	29.71	118.75	177.82
**371 Asp**	8.59	56.50	40.67	119.68	177.88
**372 Lys**	7.70	57.04	32.93	117.02	176.67
**373 Asn**	8.02	53.06	40.09	115.31	175.38
**374 Tyr**	7.74	59.87	38.26	119.47	175.82
**375 Asp**	8.88	55.05	41.36	125.14	176.57
**376 Asp**	8.31	54.38	39.97	121.40	175.91
**377 Asp**	8.09	53.93	40.95	119.51	176.22
**378 Gly**	8.86	46.69	–	113.71	174.66
**379 Ile**	7.45	60.45	40.53	119.58	174.94
**380 Thr**	8.37	60.77	69.26	120.40	174.00
**381 Tyr**	9.04	58.43	37.50	125.77	175.16
**382 Leu**	7.60	53.83	43.65	119.68	176.34
**383 Phe**	8.51	56.42	41.58	122.56	174.29
**384 Asp**	8.45	55.02	41.63	125.29	176.00
**385 Leu**	8.74	54.06	41.56	125.18	176.76
**386 Asp**	8.40	52.59	40.98	119.25	176.21
**387 Met**	8.04	56.95	31.55	118.74	175.50
**388 Phe**	8.02	57.75	41.08	116.56	174.96
**389 Asp**	8.34	54.91	41.17	120.99	176.58

**390 Asp**	8.73	53.50	40.63	123.00	176.17
**391 Ala**	8.12	53.99	18.60	123.75	178.34
**392 Ser**	7.91	60.07	62.87	113.22	174.18
**393 Glu**	7.70	56.92	27.72	118.59	174.36
**394 Tyr**	8.05	56.43	40.92	119.39	175.24
**395 Thr**	9.07	61.34	72.45	116.84	172.52
**396 Val**	9.29	61.08	33.48	122.91	174.65
**397 Asp**	9.35	52.84	43.48	128.21	175.67
**398 Ala**	8.64	50.50	19.49	128.85	176.71
**399 Gln**	9.32	59.23	28.93	118.29	176.03
**400 Asn**	8.95	53.24	40.61	112.42	174.34
**401 Tyr**	7.44	56.58	41.90	117.06	174.60
**402 Gly**	8.49	45.01	–	110.26	173.23
**403 Asp**	8.09	52.85	42.21	118.41	178.27
**404 Val**	8.56	66.59	31.66	119.66	177.89
**405 Ser**	8.20	59.68	62.29	113.08	175.59
**406 Arg**	7.51	57.59	29.68	117.91	176.47
**407 Phe**	7.93	57.34	39.86	115.83	175.38
**408 Phe**	8.03	57.56	39.22	118.62	176.24
**409 Asn**	8.38	52.60	41.20	118.65	173.74
**410 His**	8.28	55.03	30.67	117.38	174.15
**411 Ser**	7.68	57.48	64.84	113.60	173.56
**412 Cys**	9.07	59.78	28.12	119.88	174.79

**413 Ser**	8.16	55.38	63.57	114.46	172.13
**415 Asn**	8.36	52.78	40.16	116.75	174.87
**416 Ile**	7.59	59.37	42.01	112.41	173.46
**417 Ala**	9.37	50.81	23.32	121.72	175.72
**418 Ile**	8.81	61.77	38.42	120.81	176.04
**419 Tyr**	9.48	56.88	40.45	127.96	175.72
**420 Ser**	8.25	59.79	63.06	121.79	173.34
**421 Ala**	8.82	51.55	21.10	128.65	176.25
**422 Val**	9.00	59.44	35.51	117.44	175.57
**423 Arg**	8.71	57.27	32.40	121.87	176.76
**424 Asn**	8.44	52.14	37.57	118.69	175.78
**425 His**	7.73	57.09	29.17	118.92	175.67
**426 Gly**	8.53	45.89	–	105.36	173.22
**427 Phe**	8.08	56.67	40.16	119.33	175.17
**428 Arg**	7.89	56.10	28.52	123.43	176.46
**429 Thr**	8.27	63.23	69.81	112.84	174.12
**430 Ile**	7.53	60.92	40.56	119.14	173.98
**431 Tyr**	7.55	56.32	41.14	122.60	173.28
**432 Asp**	9.00	53.86	42.26	119.59	175.45
**433 Leu**	8.81	54.10	40.52	125.06	175.52
**434 Ala**	8.31	50.79	21.36	127.07	176.14
**435 Phe**	9.38	56.80	40.95	123.07	174.92
**436 Phe**	8.91	56.49	43.45	122.79	175.24

**437 Ala**	9.49	53.22	19.23	125.67	177.90
**438 Ile**	9.00	62.09	38.78	121.79	176.45
**439 Lys**	7.64	54.28	36.22	116.50	174.54
**440 Asp**	8.58	54.95	40.98	120.66	175.66
**441 Ile**	8.73	60.34	39.34	123.91	175.35
**442 Gln**	8.58	54.54	28.26	125.57	173.98
**443 Phe**	8.25	63.71	31.73	117.83	177.08
**444 Leu**	8.87	56.49	40.25	117.24	176.54
**445 Glu**	7.77	55.37	31.23	120.42	175.56
**446 Glu**	8.70	56.93	30.77	126.77	175.72
**447 Leu**	8.87	54.41	42.59	126.53	175.78
**448 Thr**	8.83	59.86	72.53	114.64	172.32
**449 Phe**	8.01	56.00	43.53	116.60	173.10
**450 Asp**	8.78	54.74	41.53	119.57	174.28
**451 Tyr**	9.07	58.87	38.63	127.76	175.49
**452 Ala**	7.95	51.49	20.26	128.38	177.35
**453 Gly**	6.42	45.49	–	106.69	174.27
**454 Ala**	8.25	52.04	18.56	125.32	177.30
**455 Lys**	7.90	55.31	32.30	117.62	176.04
**456 Asp**	8.32	54.73	41.57	116.95	175.50
**457 Phe**	8.57	59.30	39.61	122.28	175.97
**458 Ser**	8.94	57.17	63.08	115.74	173.20
**460 Val**	8.38	63.17	32.60	122.73	175.90

**461 Gln**	8.07	56.75	29.42	120.72	177.01
**473 Leu**	7.99	55.95	41.89	120.81	176.07
**474 Arg**	4.73	55.46	31.87	118.63	175.32
**475 Arg**	4.69	54.81	33.46	121.03	175.41
**476 Gln**	8.64	55.81	30.39	123.19	175.41
**477 Cys**	8.28	58.79	28.29	119.23	175.01
**478 Lys**	8.46	53.914	32.31	125.83	176.16
**479 Cys**	8.31	58.88	28.95	116.08	174.46
**480 Gly**	8.23	45.96	–	108.43	176.32
**481 Ser**	8.12	60.44	63.14	114.12	175.08
**482 Ala**	8.24	53.25	18.28	123.53	177.36
**483 Asn**	7.61	52.13	39.29	116.68	174.78
**484 Cys**	8.01	57.31	28.82	120.24	174.19
**485 Arg**	8.04	58.92	27.76	120.74	176.72
**486 Gly**	8.08	47.47	–	105.98	175.74
**487 Trp**	7.08	60.35	29.85	121.52	177.51
**488 Leu**	7.63	58.11	41.68	118.88	178.56
**489 Phe**	9.04	61.11	39.49	118.32	177.31
**490 Gly**	8.56	47.58	–	108.72	175.56

1 Simon, M. D. *et al.* The site-specific installation of methyl-lysine analogs into recombinant histones. *Cell* **128**, 1003-1012, doi:10.1016/j.cell.2006.12.041 (2007).

2 Ward, J. J., McGuffin, L. J., Bryson, K., Buxton, B. F. & Jones, D. T. The DISOPRED server for the prediction of protein disorder. *Bioinformatics* **20**, 2138-2139, doi:10.1093/bioinformatics/bth195 (2004).

3 Luger, K., Mader, A. W., Richmond, R. K., Sargent, D. F. & Richmond, T. J. Crystal structure of the nucleosome core particle at 2.8 A resolution. *Nature* **389**, 251-260, doi:10.1038/38444 (1997).
